# Self‐Immolative Polyion Complexes

**DOI:** 10.1002/marc.202500419

**Published:** 2025-07-25

**Authors:** Xueli Mei, Elizabeth R. Gillies

**Affiliations:** ^1^ Department of Chemistry The University of Western Ontario London Ontario Canada; ^2^ Department of Chemical and Biochemical Engineering The University of Western Ontario London Ontario Canada

**Keywords:** degradable, depolymerization, polyion complex, self‐immolative, stimuli‐responsive

## Abstract

Polyion complex (PICs) micelles are formed through the self‐assembly of polyelectrolytes bearing opposite charges. The ability to form PICs under fully aqueous conditions makes them attractive for the encapsulation of biopolymers such as proteins and nucleic acids for potential therapeutic applications. Stimuli‐responsive PIC micelles have the potential to release their cargo under specific biological conditions. We describe here the development of PIC micelles formed from two self‐immolative polymers (SIPs) with complementary charges. The polycationic SIP, having pendent ammonium groups, undergoes depolymerization in response to light. The polyanionic SIP, bearing pendent carboxylates and a stabilizing PEG block, undergoes depolymerization in response to a pH change from 7.4 to 6. SIP PICs composed of a 0.6 anion:cation ratio remain well dispersed at pH 7.4, but degrade at pH 6, primarily due to depolymerization of the anionic block. Irradiation with UV light leads primarily to depolymerization of the cationic block. In vitro cytotoxicity assays with C2C12 cells indicate that the PICs are quite well tolerated by the cells with low cytotoxicity up to about 0.5 mg mL^−1^. Overall, these PICs are a new platform that can potentially be used for the encapsulation and stimulus‐mediated release of ionic cargo.

## Graphical abstract

Polyion complexes (PICs) prepared by combining self‐immolative polymers (SIPs) with complementary charges are described. The polyanionic SIP is depolymerized at mildly acidic pH, while the polycationic SIP can be depolymerized upon irradiation with light. The PICs exhibit low cytotoxicity and may provide a useful platform for encapsulation and controlled release of charged cargo such as proteins and nucleic acids.

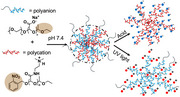

## 1 Introduction

Polyion complex (PIC) micelles are supramolecular assemblies formed by the mixing of oppositely charged polymers and/or block copolymers in aqueous solution [1]. While self‐assembly can be rationalized on the basis of electrostatic interactions between polyions of opposite charge, the main driving force is the entropically favorable release of counterions. Kataoka and coworkers reported the first example of a PIC micelle composed of poly(ethylene glycol)‐poly(L‐lysine) (PEG‐PLL) and PEG‐poly(α,β‐aspartic acid) (PEG‐P(Asp)) [2]. However, PIC micelles have been formed from a variety of different polyelectrolyte combinations including cationic block copolymers with anionic nucleic acids and cationic or anionic block copolymers with oppositely charged proteins [1]. They are particularly attractive for the encapsulation of therapeutic proteins and nucleic acids, which are often incompatible with the organic solvents commonly used in the preparation of nanoparticles from amphiphilic block copolymers [3].

In recent years, there has been growing interest in the development of stimuli‐responsive PICs [4]. For example, the mild reduction in pH from the normal extracellular environment (pH 7.4) down to pH 4.5–6.5 in the endosomal and lysosomal compartments of cells has been widely employed to facilitate the endosomal escape and release of nucleic acids from PICs by various mechanisms such as the cleavage of PEG shielding blocks linked by acetals, phosphoramidates, or imine linkers [5] or by the protonation of amino groups with carefully controlled pKa values, thereby enhancing their abilities to destabilize the endosomal membrane [6]. In another recent example, Kataoka, Cabral, and coworkers reported pH‐sensitive PICs composed of two block copolymers, including a *cis*‐aconitic anhydride‐modified PEG‐*b*‐poly(L‐lysine) and PEG‐*b*‐poly{*N*‐[*N*’‐(2‐aminoethyl)‐2‐aminoethyl]aspartamide} for the encapsulation and intracellular release of an anti‐nuclear pore complex antibody via endosomal escape [7]. Other intracellular stimuli, such as the reducing agent glutathione, which is present at 100–1000 fold higher concentrations within cells compared to the extracellular environment, have also been used to trigger hydrophobicity changes [8] or induce charge reversal in cationic polymers that were complexed with nucleic acids [9]. Combinations of multiple stimuli have also been employed. For example, PICs composed of DNA, dendritic lysine functionalized with lipids and maleic amides, and dendritic polylysine containing disulfides were designed to undergo charge conversion at mildly acidic pH through amide cleavage and protonation of the resulting amines [10]. After endosomal escape, intracellular disulfide cleavage occurred to facilitate DNA release.

A class of polymers attracting increasing attention in recent years is self‐immolative polymers (SIPs).[11] These polymers depolymerize from end‐to‐end upon a stimulus‐mediated cleavage of the polymer's end‐cap or backbone. SIPs are attractive as the stimulus to which they respond can be readily modulated by switching the end‐cap, while keeping the backbone constant. In addition, they generally undergo rapid and complete depolymerization, in contrast to other backbone‐degradable polymers. Various backbones have been developed, such as polycarbamates (PBCs) [12], polythioesters [13], poly(benzyl ether)s [14], and polyglyoxylates [15], and these backbones have been explored in applications such as adhesives, therapeutics, drug carriers, and imaging agents [16].

Thus far, there has been very little research on the incorporation of SIPs into PICs. For example, Thayumanavan and coworkers prepared PEG‐PBC block copolymers with pendent carboxylates on the PBC block and self‐assembled these SIPs with poly(diallyldimethylammonium chloride) (PDADMAC) to form a PIC [17]. Alkaline phosphatase (ALP) cleaved a phosphate end‐cap, leading to depolymerization, albeit much more slowly than in a soluble ALP‐responsive PBC. In another example, a similar PBC, but bearing a portion of hydrophobic *t*‐butyl esters and a UV light‐responsive end‐cap, was complexed with PDADMAC to form PIC vesicles encapsulating horse radish peroxidase (HRP) [18]. Light‐triggered depolymerization and release of HRP induced the gelation of tyrosine‐terminated 4‐arm‐PEG. There are also a few examples of cationic SIPs forming PICs with nucleic acids. For example, Liu and coworkers prepared hyperbranched PBCs with short chains of poly[2(dimethylaminoethyl) methacrylate] (PDMAEMA) on the periphery [19]. Depolymerization of the PBC backbone released the short PDMAEMA chains, reducing the multivalency of binding to the nucleic acids, thereby facilitating nucleic acid release. Our group recently reported polyglyoxylamides (PGAms) with pendent amino groups, which bound to nucleic acids [20]. Depolymerization under the mildly acidic conditions similar to those in the endosomal compartments facilitated nucleic acid release while converting the polymer into lower toxicity small molecule cations. However, to the best of our knowledge, there are no examples involving the combination of two SIPs with complementary charges to form PICs.

**FIGURE 1:**
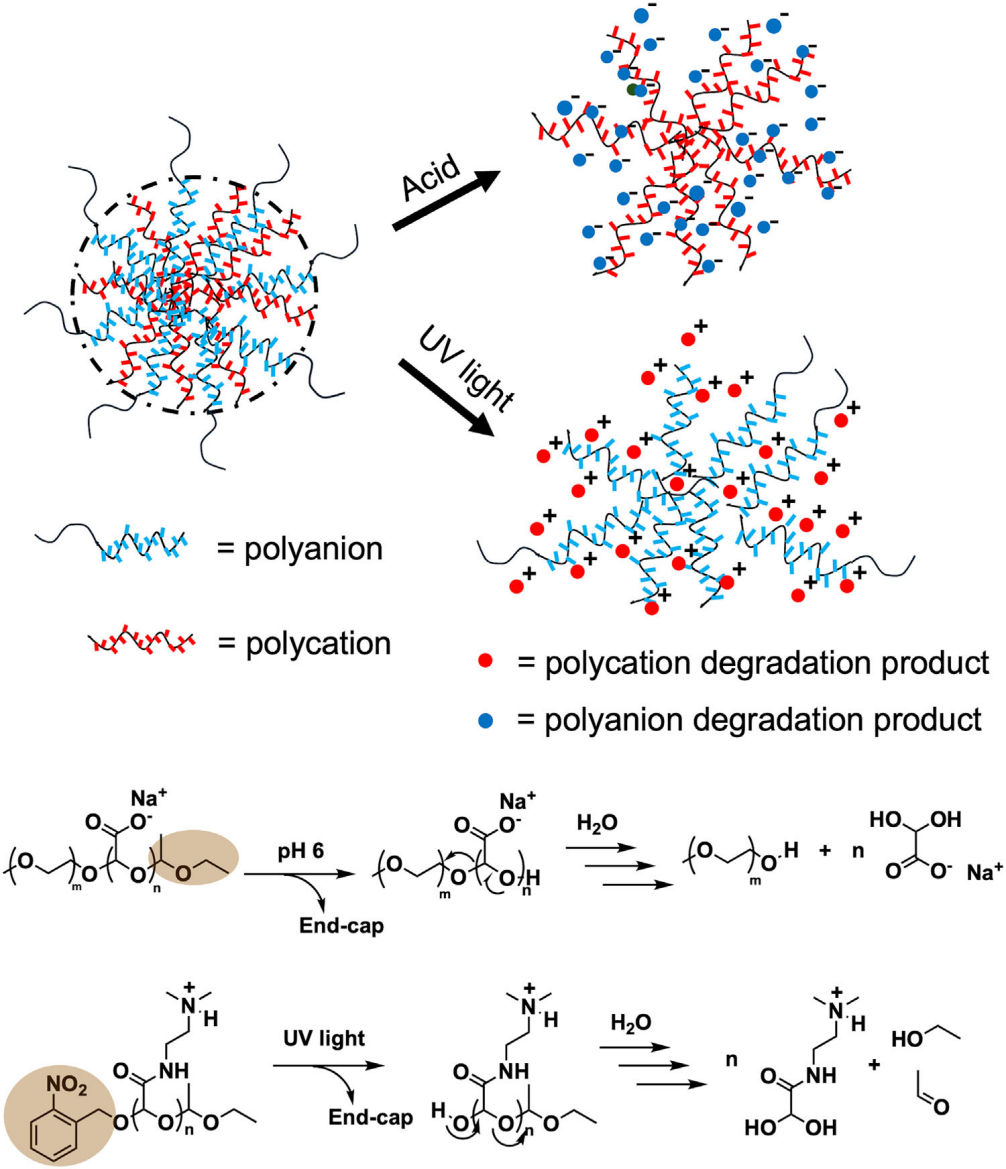
Schematic of the PIC design. PICs form by self‐assembly of self‐immolative polyanions and polycations and depolymerize upon acidification or UV light irradiation leading to PIC degradation.

Herein, we describe the development of PICs composed of a combination of anionic and cationic SIPs derived from poly(ethyl glyoxylate) (PEtG) (Figure 1). A PGAm with pendent *N*,*N*‐(dimethylamino)ethyl amide groups was selected as the cationic SIP as it can be readily synthesized by amidation and exhibited relatively low cytotoxicity compared to PGAms with other cationic pendent groups in previous work [20]. A UV light‐responsive *o*‐nitrobenzyl ether was selected for one terminus of the polymer as it can be readily cleaved in the lab with high selectivity, leading to depolymerization [21]. As the anionic SIP, poly(glyoxylic acid) (PGA) sodium salt was selected, as it can be readily synthesized by hydrolysis of PEtG under basic conditions. An acetal end‐cap was selected to enable pH‐responsive depolymerization [22]. Preliminary experiments indicated problematic aggregation of PICs composed of PGAm and PGA. Therefore, a PEG block was incorporated onto the PGA to provide steric stabilization. Self‐assembly at different anion:cation ratios was examined, and the resulting PICs were characterized by dynamic light scattering (DLS) and transmission electron microscopy (TEM). The depolymerization of the PICs in response to pH change from 7.4 to 6 and in response to light was studied. These PICs provide a potential platform for the encapsulation and stimulus‐mediated release of ionic cargo.

## 2 Results and Discussion

### 2.1 Polymer Synthesis and Characterization

**SCHEME 1:**
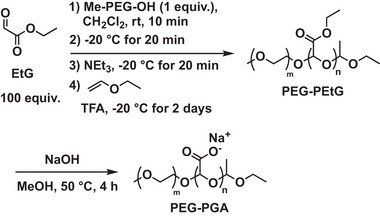
Synthesis of PEG‐PGA.

First, the anionic self‐immolative polymer was prepared. For the synthesis of PEG‐PEtG, PEG monomethyl ether (2 kg/mol) was used to initiate the polymerization of freshly distilled ethyl glyoxylate (EtG) monomer in dry CH_2_Cl_2_ in the presence of NEt_3_ as a proton transfer agent at −20°C (Scheme 1) [23]. The resulting polymer was then end‐capped by treatment with ethyl vinyl ether in the presence of trifluoroacetic acid (TFA) [24]. Successful polymerization was first confirmed by ^1^H and ^13^C NMR spectroscopic analyses, which suggested a degree of polymerization (DP_n_) of about 67, corresponding to a number average molar mass (*M_n_*) of about 6.7 kg/mol (Figures S1 and S2). Size exclusion chromatography (SEC) analysis in *N*,*N*‐dimethylformamide (DMF) relative to poly(methyl methacrylate) (PMMA) standards indicated that the PEG‐PEtG had a *M_n_* of 17.5 kg mol^−1^, and a *Ð* of 1.35 (Figure S3). Overestimation of the molar mass by SEC can likely be attributed to the larger hydrodynamic volume of PEG compared to PMMA in DMF. PEG‐PEtG was then hydrolyzed by treatment with NaOH in MeOH at 50°C to provide PEG‐PGA as the sodium salt. Unlike the starting material PEG‐PEtG, the product was fully water soluble, and ^1^H NMR spectroscopic analysis in D_2_O confirmed the successful hydrolysis based on the disappearance of the ethyl ester peaks of PEG‐PEtG in both the ^1^H and ^13^C NMR spectra (Figures S4 and S5). A comparison of the infrared (IR) spectra of PEG‐PEtG and PEG‐PGA also showed the disappearance of the carbonyl peak of the ester group at about 1750 cm^−1^ and a new peak at 1620 cm^−1^ corresponding to the carboxylate (Figure S6).

**SCHEME 2:**
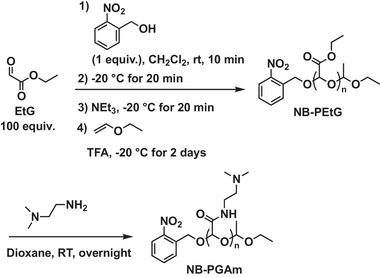
Synthesis of NB‐PGAm.

To prepare a cationic self‐immolative polymer with a UV light‐responsive terminus, the polymerization of EtG was initiated with 2‐nitrobenzyl alcohol (Scheme 2). End‐capping was again performed by treatment with ethyl vinyl ether and TFA. ^1^H and ^13^C NMR spectroscopy confirmed the structure of the resulting polymer NB‐PEtG, and a DP_n_ of about 100 was determined from the ^1^H NMR analysis, corresponding to an *M_n_* of about 10 kg/mol (Figures S7 and S8). SEC indicated that NB‐PEtG had a *M_n_* of 6.6 kg mol^−1^ and a *Ð* of 1.86 relative to PMMA (Figure S9). NB‐PEtG was treated with *N*,*N*‐dimethylethylenediamine in dioxane to provide the amine‐functionalized polymer NB‐PGAm [25]. This product was also fully water soluble. Successful amidation was determined by ^1^H NMR spectroscopic analysis, based on the disappearance of the ethyl ester peaks of NB‐PEtG at 4.21 and 1.29 ppm and the appearance of new peaks at 3.37 and 2.57 ppm corresponding to the two methylene groups on the pendant groups and at 2.25 ppm corresponding to the methyl groups on the pendent amine (Figure S10). The ^13^C NMR spectrum was also consistent with the expected structure, as the ethyl ester peaks at 62 and 14 ppm disappeared and new pendent group peaks were observed from 37 to 58 ppm (Figure S11). No significant changes were observed in the SEC profile upon converting the esters to amides (Figure S12). FT‐IR spectroscopic analysis of NB‐PGAm also showed the disappearance of the carbonyl peak of the ester groups of NB‐PEtG at about 1750 cm^−1^ and a new C═O stretching peak around 1660 cm^−1^ as well as a new N─H stretching peak around 3270 cm^−1^ corresponding to the new amide group in NB‐PGAm (Figure S13).

### 2.2 Depolymerization of PEG‐PGA and NB‐PGAm

**FIGURE 2:**
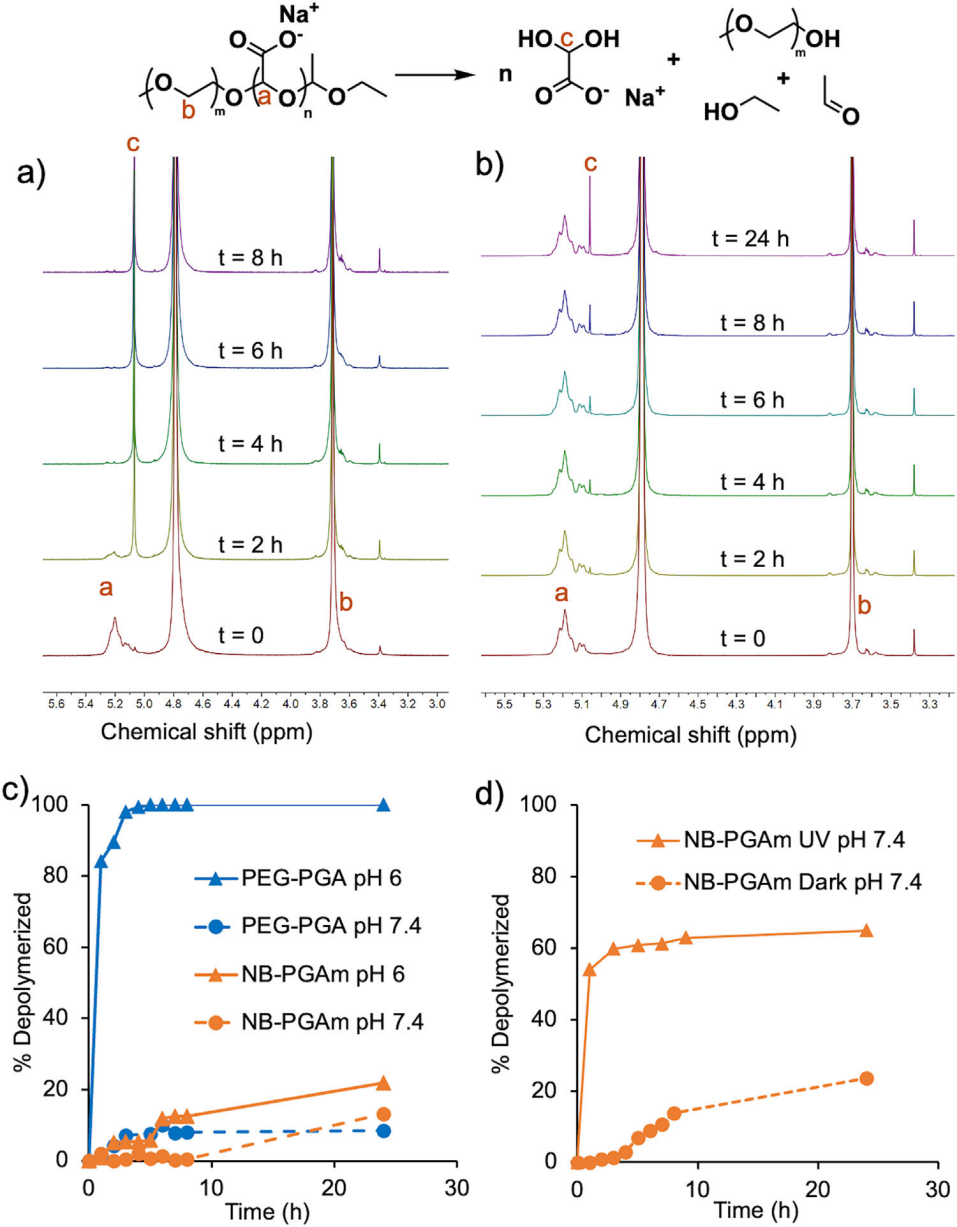
a,b) ^1^H NMR spectral overlays of PEG‐PGA over time at 37°C in deuterated PBS (400 MHz) (a) adjusted to pH 6 or (b) at pH 7.4. At pH 6, depolymerization occurred as evidenced by the disappearance of peak (a) and the appearance of peak (c) corresponding to the depolymerization product. Minimal depolymerization was observed at pH 7.4. c,d) Percent depolymerization over time for (c) PEG‐PGA and NB‐PGAm incubated at pH 6 or pH 7.4 at 37°C and (d) NB‐PGAm irradiated with UV light or kept in the dark at room temperature.

To assess the stimuli‐responsive depolymerization of PEG‐PEtG and NB‐PGAm, the polymers were treated with the stimuli corresponding to their respective end‐caps, and depolymerization was assessed by ^1^H NMR spectroscopy. For PEG‐PGA, the depolymerization was expected to be triggered by acid due to the presence of the acetal end‐cap, so depolymerization was compared at pH 6 and pH 7.4 at 37°C. The extent of depolymerization was quantified based on the integral of the remaining backbone methine peak (peak a) at 5.17 ppm compared to that of an acetonitrile internal standard at 2.1 ppm. At pH 6, complete depolymerization was observed after 4 h (Figure 2a,c), while at pH 7.4, the depolymerization reached only 8% over 24 h (Figure 2b,c). The depolymerization of NB‐PGAm was also studied at pH 6 and pH 7.4 and 37°C. Depolymerization of this PGAm was much slower, reaching only 22% at pH 6, and 13% depolymerization at pH 7.4 over 24 h (Figure 2c; Figures S14 and S15). The reason for the different pH‐responsive depolymerization behavior of PEG‐PGA and NB‐PGAm can likely be attributed to their different pendent groups. Protonation of the pendent carboxylate group adjacent to the end‐cap in PEG‐PGA may enable intramolecularly‐catalyzed cleavage of the acetal end‐cap via a 5‐membered ring hydrogen‐bonded species, whereas this mechanism isn't possible for the NB‐PGAm. Therefore, PEG‐PGA was much more sensitive to pH.

The depolymerization of NB‐PGAm with and without UV light irradiation was studied in pH 7.4 deuterated phosphate‐buffered saline (PBS) at 20°C (Figure 2d). The control sample was kept in the dark while the irradiated sample was exposed to a 400 W mercury lamp in a photochemical reactor. The irradiated polymer depolymerized about 65% during the first 2 h, after which depolymerization plateaued (Figure S16a). The non‐irradiated polymer depolymerized only 23% (Figure S16b). This 23% depolymerization can be attributed to the gradual cleavage of the acetal end‐cap from NB‐PGAm, as reported above. The plateau in depolymerization of the irradiated polymer can likely be attributed to incomplete initiation of the polymer with 2‐nitrobenzyl alcohol, an issue that we have previously reported [21]. Nevertheless, NB‐PGAm exhibited clear, photo‐responsive depolymerization. Overall, the depolymerization studies indicated that mildly acidic pH could trigger depolymerization of PEG‐PGA, while UV light could trigger depolymerization of NB‐PGAm.

### 2.3 Preparation and Characterization of PICs

**FIGURE 3:**
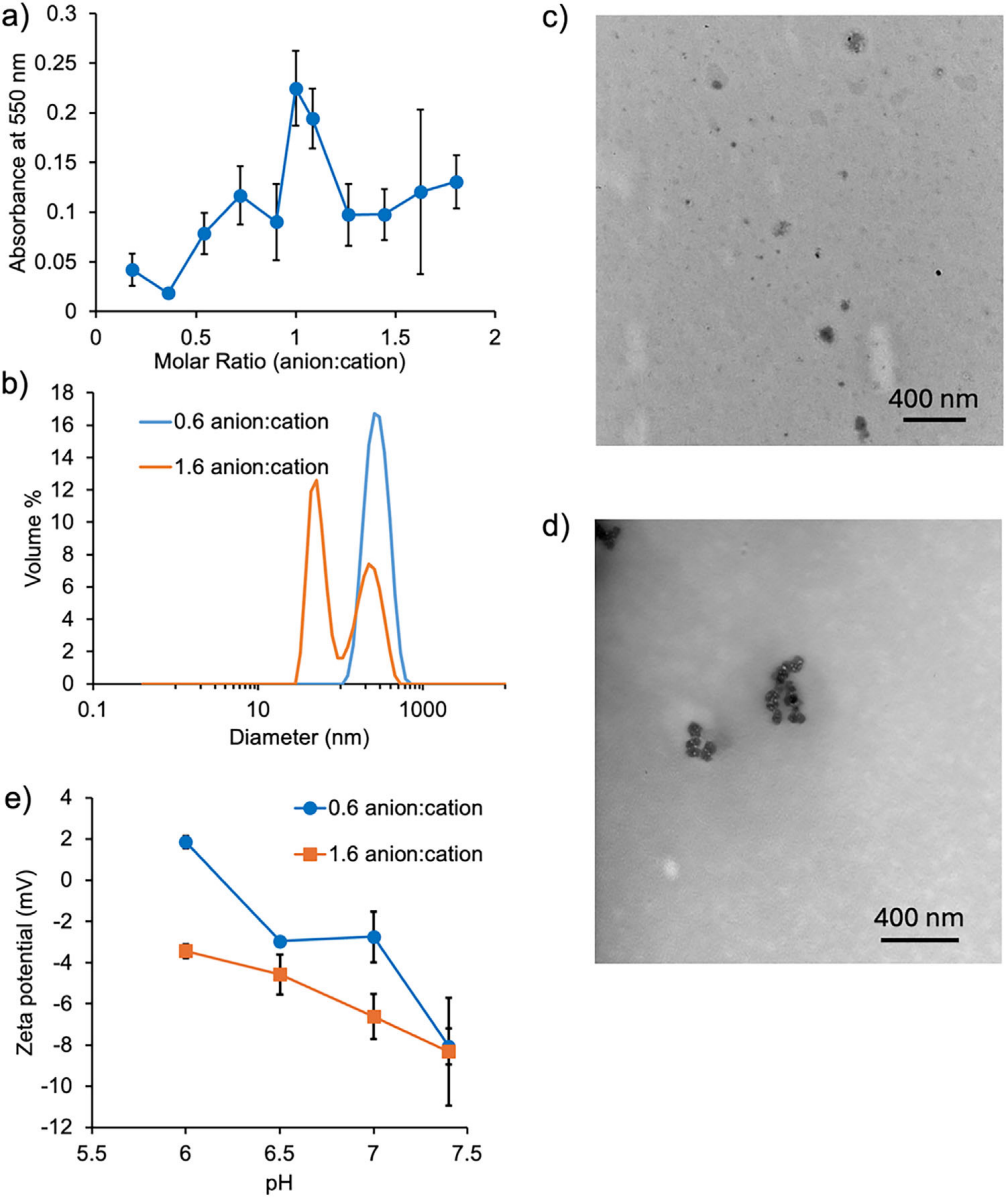
Characterization of PICs formed from PEG‐PGA and NB‐PGAm: a) Turbidity (indicated by absorbance at 550 nm) of PIC suspensions prepared at 2.5 mg mL^−1^ in pH 7.4 PBS at varying anion:cation ratios. Each ratio was evaluated in triplicate and the error bars correspond to the standard deviations; b) Representative volume distributions for PICs formed from 0.6 and 1.6 anion:cation ratios measured by DLS; c‐d) Representative TEM images of PICs prepared at c) 0.6 and d) 1.6 anion:cation ratios; e) Zeta‐potentials for PICs prepared from 0.6 and 1.6 anion:cation ratios, measured in PBS adjusted to different pH values. Each sample was evaluated in triplicate, and the error bars correspond to the standard deviations.

To find suitable complexation conditions for the PICs, various anion:cation ratios from 0.2 to 1.8 were initially screened based on the absorbance at 550 nm as a measure of turbidity. Since the system was designed for potential biomedical applications, pH 7.4 PBS was selected as the solvent. The turbidity increased as the anion:cation ratio was increased from 0.4 to 0.7, and peaked at a 1:1 ratio, which was expected due to charge neutralization of the resulting assemblies and consequent aggregation (Figure 3a). From a 1:1.2 ratio and beyond, the turbidity decreased and then remained relatively constant. As the goal was to obtain nanoparticles as opposed to larger aggregates, lower turbidity was desired. Consequently, the 0.6 and 1.6 ratios were selected for further study. Based on DLS, the volume distribution for the 0.6 anion:cation ratio was monomodal with a peak diameter of 275 ± 19 nm (Figure 3b). TEM showed particles with diameters on the order of 100–200 nm (Figure 3c). For the 1.6 ratio, the PICs had a bimodal distribution of diameters, with one volume distribution peak at 32 ± 16 nm and the other one at 263 ± 63 nm. This distribution likely corresponded to a population of smaller nanoparticles and their aggregates, which was also supported by TEM (Figure 3d).

The zeta‐potential values of the PICs were also measured across a pH range of 6–7.4 at 0.6 and 1.6 anion:cation ratios. For the 0.6 anion:cation ratio, as the pH was reduced from 7.4 to 6, the zeta‐potential increased from −8 to +2. The tendency of the PICs to have relatively low and negative zeta‐potential may arise from their PEG shell, which is known to impart mildly negative zeta‐potential [26]. In addition, it is likely that not all the pendant *N*,*N*‐(dimethylamino)propyl amide groups on NB‐PGAm were protonated at pH 7.4, yielding fewer cations compared to the stoichiometric number of amines. Previous research has shown that the pK_a_ value for poly[*N*,*N*‐(dimethylamino)ethyl methacrylate], which also has tertiary amine pendent groups, is in the range of 6.4–7.4 depending on the conditions [27]. Unfortunately, attempts to measure the specific pK_a_ values for PEG‐PGA and NB‐PGAm are complicated by their depolymerization at acidic pH during titration. For the anion:cation ratio of 1.6, the zeta‐potential increased from −8 to about −3.5. Thus, overall, the 1.6 anion:cation ratio PICs always had a negative zeta‐potential, whereas the zeta‐potential of the 0.6 anion:cation ratio PICs was negative down to pH 6.5 and then became slightly positive at pH 6.

### 2.4 PIC Depolymerization Triggered by Light and pH Change

**FIGURE 4:**
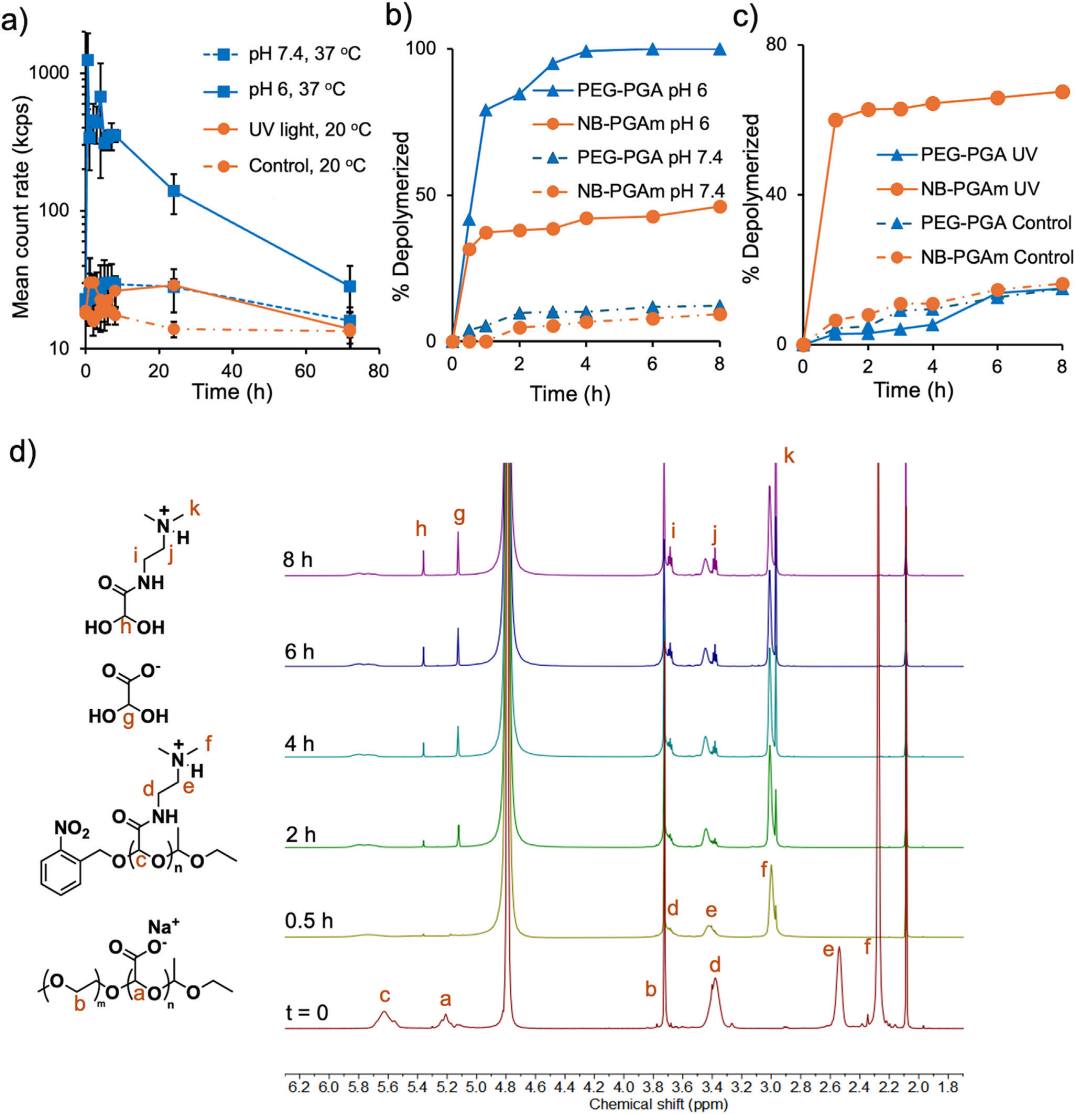
Depolymerization results for PICs composed of a 0.6 anion:cation ratio: a) Mean derived count rate versus time for PICs incubated at 37°C at either pH 7.4 or 6 and at pH 7.4 either with or without UV light irradiation (20°C); b,c) Percent depolymerization versus time for the PEG‐PGA and NB‐PGAm components of PICs, as measured by ^1^H NMR spectroscopy for: b) PICs incubated at pH 6 or 7.4 (37°C); c) PICs irradiated with UV light or kept in dark at 20°C at pH 7.4. d) ^1^H NMR (400 MHz) spectral overlay of PICs in deuterated PBS (pH 7.4) containing 0.1% acetonitrile before (t = 0) and after pH adjustment to 6, followed by incubation at 37°C for various time periods.

Depolymerization studies focused on PICs prepared using a 0.6 anion:cation ratio, as the DLS and TEM results suggested that they formed well dispersed rather than aggregated particles at pH 7.4. To assess the overall changes in aggregation or dissociation of the PICs during the degradation process, PICs subjected to mildly acidic pH or irradiation with UV light were first monitored by DLS. The mean derived count rate was measured as an indicator of the number of scattering nanoparticles and their diameters. The PICs were prepared in pH 7.4 PBS at 2.5 mg mL^−1^ and a 0.6 anion:cation ratio. They were relatively stable at pH 7.4 and 37°C over 72 h (Figure 4a; Figure S17). However, after adjusting the suspension to pH 6, the mean count rate immediately increased about 10‐fold, and the suspension became turbid, indicating aggregation (Figure S18). This aggregation likely arose from the transition through zero zeta potential, where the nanoparticle suspension was very unstable. However, as depolymerization proceeded over the next 48 h, the turbidity decreased substantially, and the count rate also decreased. After 72 h, the DLS volume distribution showed only a sub‐10 nm diameter distribution of particles (Figure S17). Upon UV light irradiation at 20°C, which was expected to trigger depolymerization of the NB‐PGAm, only minor perturbations in the count rate were observed. This observation can likely be attributed to some residual undepolymerized polycation as discussed above, which held together some proportion of PICs or aggregates. However, again, only a sub‐10 nm distribution of particles was observed in the volume distribution by DLS after 72 h (Figure S17). The control non‐irradiated PICs also exhibited only minor changes in count rate over 72 h.

The molecular‐level depolymerization was also studied by ^1^H NMR spectroscopy. The PICs were prepared in deuterated pH 7.4 PBS at 10 mg/mL and the anion:cation mixing ratio of 0.6. To assess the effect of pH, they were then held at pH 7.4 or adjusted to pH 6 and incubated at 37°C. Even when incorporated into the PIC, the PEG‐PGA depolymerized rapidly at pH 6, with ≈80% depolymerization in 1 h (Figure 4b,d). After about 4 h, the PEG‐PGA had fully depolymerized. Meanwhile, the NB‐PGAm underwent about 40 % depolymerization over 8 h. At pH 7.4, less than 15% depolymerization was observed for both PEG‐PGA and NB‐PGAm over 8 h (Figure S19). Overall, these results were in good agreement with those for the non‐complexed polymers.

In the UV light‐induced degradation study performed at pH 7.4 and 20°C, the NB‐PGAm component depolymerized rapidly, reaching 60% degradation after 1 h of irradiation and 70% after 8 h (Figure 4c; Figure S20). In contrast, the PEG‐PGA remained quite stable, with only ≈10% depolymerization after 8 h. When stored in the dark for 8 h, both PEG‐PGA and NB‐PGAm remained quite stable, undergoing only ≈10 % depolymerization after 8 h (Figure S21). These results generally aligned with the individual polymer depolymerization study data as well. Overall, these results indicated that we can quite selectively depolymerize either one of the polyelectrolyte components in the PICs.

### 2.5 In Vitro Cytotoxicity Assay

**FIGURE 5:**
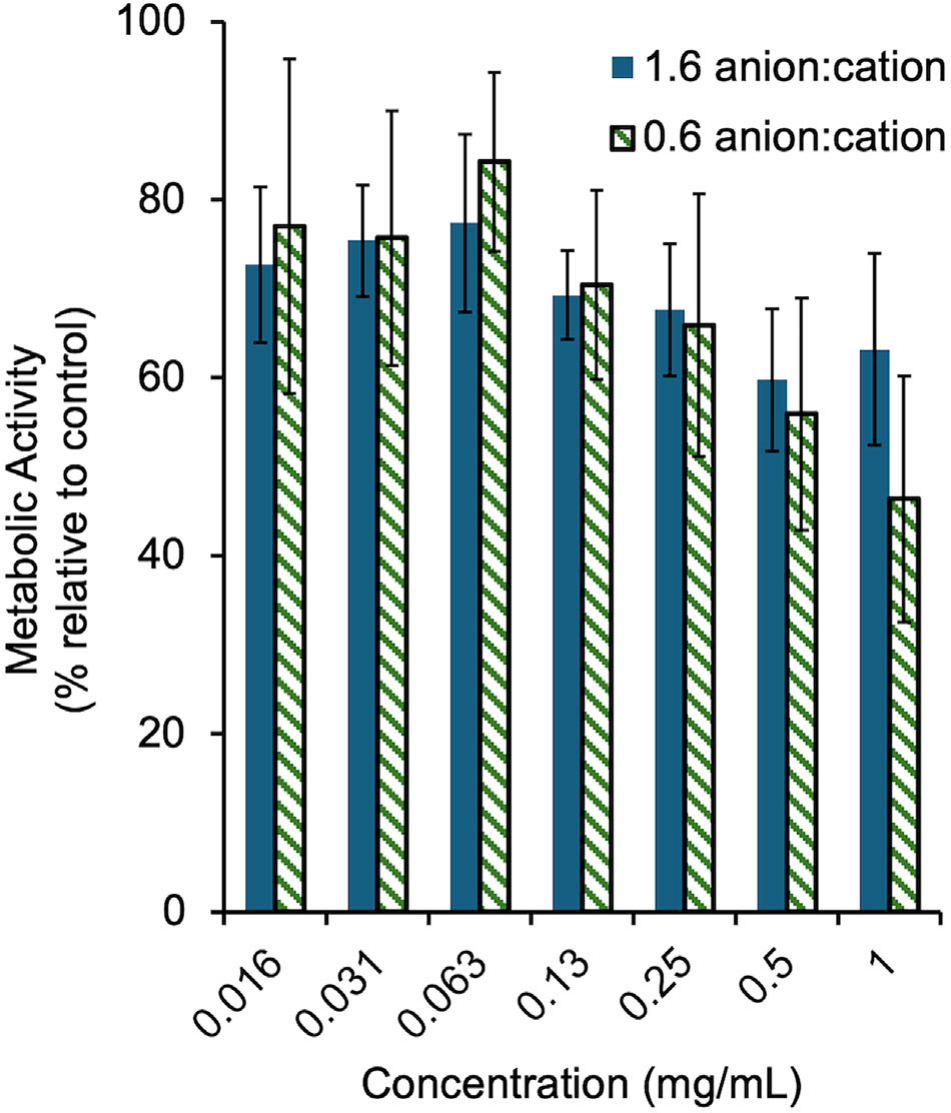
Metabolic activity of C2C12 cells exposed to varying concentrations of PICs (0.6 or 1.6 anion:cation ratio) as assessed by an MTT assay. Error bars correspond to the standard deviation on six replicates.

To determine the potential toxicity of the PICs, in vitro cell toxicity studies were performed on a mouse myoblast cell line, C2C12. This cell line has been commonly used to evaluate the toxicity of biomaterials [28]. PICs prepared at 0.6 and 1.6 anion:cation ratios were added to cells at concentrations ranging from 16 µg mL^−1^ to 1.0 mg mL^−1^. Based on a 3‐(4,5‐dimethylthiazol‐2‐yl)‐2,5‐diphenyl tetrazolium bromide (MTT) assay, concentrations up to 0.5 mg mL^−1^ were quite well tolerated, with metabolic activities over 60% relative to the control (Figure 5). At 1 mg mL^−1^, PICs composed of excess polyanion (1.6 anion:cation ratio) still retained greater than 60% metabolic activity of the control, while those composed of excess polycation (0.6 anion:cation ratio) had a trend towards higher cytotoxicity with about 50% metabolic activity. However, the differences between the two systems were not statistically significant. These trends were expected, as it is well documented that polycations can induce cell membrane damage at higher concentrations [29]. It is worth noting that the cell viability below that of the control, even at the lowest concentrations (16–63 µg mL^−1^) is somewhat perplexing, as there is no obvious concentration‐dependence in this range. However, our group has observed the same phenomenon in studies on this cell line previously [28c]. Overall, this cytotoxicity study showed that the PICs prepared had modest effects on cell viability, making them promising for further investigations in the area of drug encapsulation and stimulus‐mediated release.

## 3 Conclusion

Stimuli‐responsive PICs composed of two different self‐immolative polymers, PEG‐PGA and NB‐PGAm, were prepared and studied. PEG‐PGA depolymerized in response to mildly acidic pH (pH 6), while NB‐PGAm depolymerized in response to UV light, while also exhibiting slow depolymerization under mildly acidic conditions. A complexation study based on turbidity analysis was conducted to screen anion:cation mixing ratios, and 0.6 and 1.6 anion:cation ratios were selected for further experiments. DLS measurements showed that the PICs prepared at a 0.6 anion:cation ratio exhibited a monomodal diameter distribution with a peak diameter of 275 ± 19 nm, while the assemblies formed from the 1.6 ratio had a multimodal diameter distribution with peaks at volume distribution peaks of 32 ± 16 nm and 263 ± 63 nm. These results were supported by TEM imaging. Depolymerization of the PICs in response to pH change and UV light irradiation was studied by ^1^H NMR spectroscopy and DLS measurements. The results indicated that a pH change from 7.4 to 6 induced rapid aggregation of the PICs, followed by depolymerization mainly of the PEG‐PGA. On the other hand, UV light primarily induced depolymerization of the NB‐PGAm, while some assemblies remained intact based on DLS. Cytotoxicity studies with C2C12 cells indicated that the PICs exhibited only modest cytotoxicity, with a trend towards lower cytotoxicity for the more anionic PICs (1.6 anion:cation ratio). This self‐immolative PIC system could serve as a promising platform for the loading and stimulus‐mediated release of cargo such as protein or nucleic acid therapeutics. For such applications, the small pH change can serve as a viable trigger, while the end‐cap responsive to UV light should be replaced with an end‐cap responsive to tissue penetrating near‐IR light or other intrinsic biological stimuli.

## 4 Experimental Section

### 4.1 General Materials

MTT and EtG in toluene solution (50% w/w) were obtained from Alfa Aesar (Ward Hill, MA, USA). Toluene, *n*‐pentane, dimethyl sulfoxide (DMSO), and NaOH were obtained from Fisher Scientific (Mississauga, Canada). Triethylamine (NEt_3_), ethyl vinyl ether, dichloromethane, dioxane, NaCl, PEG monomethyl ether (*M_n_* = 2 kg mol^−1^), sodium bicarbonate, TFA), CDCl_3_ (99.8 atom% D), and D_2_O (99.9 atom% D) were obtained from Millipore Sigma (Oakville, Canada). NaH_2_PO_4_ and Na_2_HPO_4_ were obtained from Caledon Laboratories Ltd. (Georgetown, Canada). Sodium carbonate was purchased from Bioshop Canada Inc. (Burlington, Canada). Dulbecco's Modified Eagle Medium (DMEM), fetal bovine serum (FBS), Glutamax, and penicillin/streptomycin were obtained from VWR (Mississauga, Canada). Dialysis membranes and centrifugal dialysis devices were purchased from Spectrum Laboratories Inc. (New Jersey, USA). PBS containing 20 mM Na_2_HPO_4_, 5 mM NaH_2_PO_4_, and 120 mM NaCl was prepared from deionized water. The pH was adjusted to 7.4 with concentrated NaOH while monitoring with a pH meter. Water used to prepare the buffer solutions was obtained from a Barnstead Easypure II system with a measured resistivity of 15 MΩ or greater. Under a nitrogen atmosphere, toluene was distilled over sodium, while NEt_3_ and CH_2_Cl_2_ were distilled over CaH_2_. EtG was purified by distillation over P_2_O_5_ as previously reported [30]. All other chemicals were used as received.

### 4.2 General Procedures

NMR spectroscopy was conducted on a Bruker AvIII HD 400 MHz Spectrometer (^1^H 400.09 MHz, ^13^C 100.5 MHz). SEC was performed in DMF containing 10 mM LiBr and 1% (v/v) NEt_3_. The instrument was composed of a Waters 515 HPLC pump, Waters In‐Line Degasser AF, a Waters Temperature Control Module II equipped with two PLgel mixed‐D 5 µm (300 ×1.5 mm) columns attached to a corresponding PLgel guard column, and a Wyatt Optilab Rex RI detector. Samples were dissolved in the mobile phase at ≈5 mg/mL and filtered through a 0.2 µm polytetrafluoroethylene (PTFE) syringe filter prior to injection using a 50 µL loop. Samples were run at a flow rate of 1 mL min^−1^ for 30 min at 85°C. The *M_n_*, *M_w_*, and *Đ* were determined relative to PMMA standards.

### 4.3 Synthesis of PEG‐PEtG

1.71 g (0.86 mol, 1.0 equiv.) of PEG (2 kg mol^−1^) was added to a 250 mL round bottom flask, and then 60 mL of dry toluene was added. The mixture was heated at reflux (112°C) with a Dean‐Stark trap to remove water from the PEG. After 4 h, the temperature was raised to 114°C to remove excess toluene until the volume was ≈20 mL. The solution was then carefully added to a flame‐dried 100 mL Schlenk flask. Then, 8.0 mL of freshly distilled EtG (86 mmol, 100 equiv.) was rapidly added to the flask under high N_2_ flow. The mixture was stirred for 30 min at room temperature before cooling the solution to −20°C using an ethylene glycol dry ice bath and stirring at that temperature for 30 min. Then, 0.27 mL of freshly distilled NEt_3_ (1.9 mmol, 3.0 equiv.) was added, and the resulting solution was stirred for 20 min before the addition of ethyl vinyl ether (0.37 mL, 3.9 mmol, 6.0 equiv.). After 5 min of stirring, 0.6 mL of TFA was added to the reaction mixture, and it was stirred for another 10 min. The resulting reaction mixture was then kept in a −20°C freezer for 2 days to allow the end‐capping reaction to proceed to completion. Then, another 0.27 mL of freshly distilled NEt_3_ (1.9 mmol, 3.0 equiv.) was added to the flask, and it was gradually warmed to room temperature. The polymerization mixture was concentrated in vacuo at 45°C to yield a crude residue, and then the residue was re‐dissolved in 10 mL of CH_2_Cl_2_ and added dropwise to a vigorously stirring methanol/water mixture (5/1; 250 mL). During this process, the pH of the solution was carefully maintained at pH 8–9 by the addition of 0.1 M NaOH. After storage overnight at −20°C, the solvent was decanted, and the resulting polymer was collected by redissolving in CH_2_Cl_2_ and drying in vacuo. Yield = 2.8 g, 26%.^1^H NMR (CDCl_3_, 400 MHz, δ): 5.77−5.41 (m, 67H), 4.21 (br s, 128H), 3.64 (s, 176H), 1.29 (br s, 203H). ^13^C{^1^H} NMR (CDCl_3_, 100 MHz, δ): 165.7, 93.2, 70.7, 62.2, 14.0. FT‐IR: 1748, 2880, 2982 cm^−1^. SEC (DMF, PMMA): *M_n_* = 17.5 kg mol^−1^, *M_w_* = 23.6 kg mol^−1^, *Ð* = 1.35.

### 4.4 Synthesis of PEG‐PGA

NaOH (250 mg, 6.28 mmol, 3.0 equiv.) was dissolved in 3.0 mL of MeOH at 50°C. Then, PEG‐PEtG (200 mg, 2.09 mmol of ester, 1.0 equiv.) was added. After 4 h of stirring, the reaction mixture was gradually cooled to room temperature and centrifuged at 3000 rpm for 5 min. The supernatant was carefully decanted, and the precipitate was redissolved in DI water. The solution was dialyzed against a 2 kDa molecular weight cut‐off (MWCO) regenerated cellulose membrane in pH 9 deionized water (adjusted using 2 m aqueous NaOH) overnight and then lyophilized to give a white solid. Yield = 78 mg, 48%. ^1^H NMR (D_2_O, 400 MHz, δ): 5.18 (br s, 84H), 3.92−3.44 (s, 176H), 3.30 (s, 4H) 1.25 (br s, 3H), 1.06 (br s, 3H).^13^C{^1^H} NMR (D_2_O, 100 MHz): δ 172.9, 95.0, 69.6. FT‐IR: 1619, 2880 cm^−1^.

### 4.5 Synthesis of NB‐PEtG

In a flame‐dried 100 mL Schlenk flask, 2‐nitrobenzyl alcohol (98.5 mg, 0.64 mmol, 1.0 equiv.) was dissolved in 20 mL of dry CH_2_Cl_2_ at room temperature. Then, 6.0 mL of freshly distilled EtG (64 mmol, 100 equiv.) was rapidly added to the flask under high N_2_ flow. The solution was stirred for 30 min at room temperature before cooling to −20°C using an ethylene glycol dry ice mixture bath and stirring at that temperature for 30 min. Then, 0.27 mL of freshly distilled NEt_3_ (1.9 mmol, 3.0 equiv.) was added, and the resulting solution was stirred for 20 min before the addition of ethyl vinyl ether (0.37 mL, 3.9 mmol, 6.0 equiv.). After 5 min, 0.6 mL of TFA was added to the mixture and it was stirred for another 10 min. The resulting mixture was then kept in a −20°C freezer for 2 days to allow the end‐capping reaction to go to completion. Then, another 0.27 mL of freshly distilled NEt_3_ (1.9 mmol, 3.0 equiv) was added to the flask, and the reaction mixture was allowed to gradually warm to room temperature. The polymerization mixture was then concentrated in vacuo at 45°C to yield a crude residue. The residue was redissolved in 10 mL of CH_2_Cl_2_ and added dropwise to a vigorously stirring methanol/water mixture (5/1; 250 mL). During this process, the pH of the solution was carefully maintained at pH 8–9 by the addition of 0.1 m NaOH as needed. The beaker was then sealed and stored in a −20°C freezer. The next day, the solvent was decanted, and the resulting polymer was collected by redissolving in CH_2_Cl_2_ and drying in vacuo. Yield = 3.9 g, 60%. ^1^H NMR (CDCl_3_, 400 MHz, δ): 8.07 (br s, 1H), 7.86 (br s, 1H), 7.65 (br s, 1H), 7.44 (br s, 1H), 5.99−5.34 (br m, 104H), 4.21 (br s, 210H), 1.29 (br s, 330H).^13^C{^1^H} NMR (CDCl_3_, 100 MHz, δ): 166.6−164.9, 93.9−91.7, 62.1, 13.9. FT‐IR: 2986, 1748 cm^−1^. SEC (DMF, PMMA): *M_n_* = 6.6 kg mol^−1^, *M_w_* = 12.2 kg mol^−1^, *Ð* = 1.86.

### 4.6 Synthesis of NB‐PGAm

NB‐PEtG (0.20 g, 2.9 mmol of ester, 1.0 equiv.) was dissolved in 3 mL of 1,4‐dioxane in a 20 mL glass vial. *N,N*‐Dimethylethylenediamine (0.96 mL, 8.8 mmol, 3.0 equiv) was added to the solution, and the vial was sealed, protected from light, and stirred overnight at room temperature. The reaction mixture was then concentrated and precipitated in 10 mL of *n*‐pentane twice to afford 0.13 g of a clear, light yellow, brittle solid. Yield = 57%. ^1^H NMR (D_2_O, 400 MHz, δ): 8.15 (br s, 1H), 7.78 (br, s, 2H), 7.63 (br s, 1H), 5.61 (br s, 49H), 3.37 (br s, 91H), 2.52 (br s, 94H), 2.25 (br s, 263H), 1.37 (br s, 3H), 1.16 (br s, 3H).^13^C{^1^H} NMR (D_2_O, 100 MHz, δ): 167.1, 89.0, 66.6, 57.8, 56.7, 44.2, 43.7, 36.9. FT‐IR: 1545, 1665, 2775, 2945, 3270 cm^−1^. SEC (DMF, PMMA): *M_n_* = 6.4 kg mol^−1^, *M_w_* = 12.2 kg mol^−1^, *Ð* = 1.92.

### 4.7 Depolymerization of PEG‐PGA and NB‐PGAm

PEG‐PGA or NB‐PGAm was dissolved in pH 7.4 deuterated PBS at a concentration of 10 mg/mL.

For the experiments investigating the effect of pH, the control group was kept at 37°C at pH 7.4. For the acid triggered depolymerization samples, the pH value was carefully adjusted to pH 6 using 0.1 m HCl in D_2_O. After vortexing for 5 s, the ^1^H NMR spectrum was immediately obtained, and then the sample was incubated at 37°C. NMR spectra were subsequently obtained at various timepoints, and between measurements, the samples were stored at 37°C.

For the UV‐light triggered depolymerization study, the control group was kept at 20°C in the dark. For the UV light‐irradiated sample, the NMR tube was kept at 20°C in a Photochemical Reaction Cabinet from Ace Glass Incorporated (Vineland, NJ, USA), equipped with a 400 W Mercury bulb. The bulb provided the following energy densities: UVA (320–400 nm, 9999 mJ/cm^2^), UVB (290–320 nm, 9462 mJ cm^−2^), UVC (200–290 nm, 2016 mJ cm^−2^), and UVV (395–445 nm, 6066 mJ cm^−2^). These measurements were taken using a PP2‐H‐U Power Puck II from EIT Instrument Markets (Sterling, VA, USA). NMR spectra were obtained at various timepoints, and then the samples were returned to the Photochemical Reaction Cabinet or stored in the dark.

### 4.8 Polymer Complexation Study

To screen for suitable complexation conditions, a series of different anion:cation ratios was tested. PEG‐PGA and NB‐PGAm were dissolved separately in pH 7.4 PBS to afford 2.5 mg mL^−1^ stock solutions. To a 5 mL vial containing a stir bar, the PEG‐PGA solution was added first, and then the NB‐PGAm solution was added dropwise with gentle stirring at the target ratio. The resulting suspension was stirred at room temperature for 20 min while protected from light. 200 µL of each suspension was added to one well of a 96‐well plate. Turbidity of the different suspensions was then determined by measurement of the absorbance value at 550 nm using a plate reader (Tecan Infinite M1000 Pro). Each ratio was prepared and evaluated in triplicate. The data are reported as the mean ± standard deviation.

### 4.9 Dynamic Light Scattering and Zeta‐Potential Measurements of PICs

PICs were prepared as described at the concentration of 2.5 mg mL^−1^ and anion:cation ratios of 0.6 and 1.6. DLS measurements were conducted on a Zetasizer Nano ZS instrument (Malvern Instruments Ltd, Malvern, UK) at room temperature. To a disposable low‐volume cuvette, ≈500 µL of PIC suspension was added. After the size measurements, the pH of the solution was measured with pH paper, and then the zeta potential value of each sample was measured using the same instrument. The pH value was then adjusted carefully using 0.1 m HCl aq. to 7, 6.5, and 6. Zeta potential values of the samples at each pH value were obtained. Each anion:cation ratio was tested in triplicate.

### 4.10 Transmission Electron Microscopy of PICs

PEG‐PGA and NB‐PGAm were dissolved separately in 0.001 m NaCl aq. to obtain 2.5 mg mL^−1^ solutions. By carefully adding the NB‐PGAm solution dropwise to the PEG‐PGA solution, PICs with anion:cation ratios of 0.6 and 1.6 were prepared separately. After stirring at room temperature for 20 min, a drop of each PIC suspension was placed on a nickel TEM grid (Electron Microscopy Science, Hatfield, US) and allowed to sit for 5 min before wicking away the excess liquid. The grids were then allowed to dry overnight with protection from light. Each grid was loaded into the microscope and imaged by a Philips CM10 (Philips, Amsterdam) using an acceleration voltage of 80 kV.

### 4.11 DLS Monitored Depolymerization Studies of the Polyion Complexes

2.5 mg mL^−1^ stock solutions of NB‐PGAm and PEG‐PGA were prepared in pH 7.4 PBS. By adding the NB‐PGAm solution dropwise with stirring to the PEG‐PGA solution at the anion:cation ratio of 0.6, 4 mL of a 2.5 mg mL^−1^ PIC suspension was obtained. The solution was stirred for 20 min and then divided into four 5 mL glass vials. At each time point, the PIC suspension was transferred into a 0.5 mL disposable cuvette, and the mean count rate was measured by DLS while fixing the attenuator (index = 7).

#### 4.11.1 Acid Triggered Degradation

After taking the initial DLS measurement (t = 0), the control sample was stored at 37°C protected from light, while the pH of the acid‐triggered samples was carefully adjusted to 6 using 0.1 m HCl in D_2_O while stirring. Then, DLS measurements were obtained for both samples again (t = 0.5 h), and the vials were stored at 37°C protected from light. At each time point, DLS measurements were obtained, and then the samples were returned to storage in the dark at 37°C with stirring.

#### 4.11.2 UV Light Triggered Degradation

After obtaining an initial DLS measurement (t = 0), the control sample was stored at 20°C in the dark. For the UV light‐irradiated sample, the vial was kept at 20°C in a Photochemical Reaction Cabinet as described above. At each time point, DLS measurements were obtained, and then the samples were returned to storage in the dark or in the Photochemical Reaction Cabinet at 20°C.

### 4.12 NMR Spectroscopy Depolymerization Studies of the Polyion Complexes

NB‐PGAm and PEG‐PGA were dissolved separately in pH 7.4 deuterated PBS to obtain 10 mg mL^−1^ solutions. By adding the NB‐PGAm solution dropwise with stirring to the PEG‐PGA solution at the anion:cation ratio of 0.6, 4 mL of a 10 mg mL^−1^ PIC suspension was obtained. 1 µL mL^−1^ of acetonitrile was added as an internal standard. The solution was stirred for 20 min and then divided into four NMR tubes.

#### 4.12.1 Acid Triggered Degradation

After obtaining initial ^1^H NMR spectra (t = 0), the control sample was stored at 37°C (in the dark), while the pH of the acid‐triggered sample was carefully adjusted to 6 using 0.1 m HCl in D_2_O. Then, ^1^H NMR spectra were obtained for both groups (t = 0.5 h) and the tubes were stored in the dark at 37°C. At each time point, ^1^H NMR spectra were obtained and then the samples were returned to storage in the dark at 37°C.

#### 4.12.2 UV Light Triggered Degradation

After obtaining initial ^1^H NMR spectra (t = 0), the control sample was stored at 20°C in the dark. For the UV light‐irradiated sample, the NMR tube was kept at 20°C in a Photochemical Reaction Cabinet as described above. At each time point, ^1^H NMR spectra were obtained and then the samples were returned to storage in the dark or in the Photochemical Reaction Cabinet at 20°C.

### 4.13 In Vitro Cytotoxicity Study

PICs were prepared at 0.6 and 1.6 anion:cation ratios at a concentration of 10 mg mL^−1^ as described above, but in a biosafety cabinet using sterilized pH 7.4 PBS. C2C12 mouse myoblast cells were plated in a 96‐well plate (Corning Flat Bottom Plate) at a density of 5000 cells per well and cultured in DMEM containing 10% FBS, 1% Glutamax (100×) solution, and antibiotics (100 units mL^−1^ of each of Streptomycin and Penicillin) for 24 h in an atmosphere containing 5% CO_2_ at 37°C. Then, the medium was removed and replaced with PIC suspensions with varying concentrations ranging from 0.016–1 mg mL^−1^. 0.2–0.05 mg mL^−1^ sodium dodecyl sulfate was used as a positive control, and fresh medium was used as a negative control. The cells were then incubated at 37°C for 48 h. After incubation, the medium was again aspirated. MTT was dissolved in cell culture media at a concentration of 0.5 mg mL^−1^, and 110 µL of the MTT media was added to each well, before being incubated for 4 h at 37°C. The media was then aspirated, and 50 µL of DMSO was added to each well to solubilize the purple crystals. The plate was then placed in a plate reader (Tecan Infinite M1000 Pro), and the absorbance at 540 nm was measured to quantify the relative metabolic activities of the cells. Six technical replicates were performed. The data is reported as the mean ± standard deviation.

## Conflicts of Interest

The authors declare no conflicts of interest.

## Supporting information




**Supporting file**: marc70005‐sup‐0001‐SuppMat.pdf

## Data Availability

The data that support the findings of this study are available in the supplementary material of this article.
