# Supplementary material for: Self‐Immolative Polyion Complexes
Source: Macromol Rapid Commun. 2025 Jul 25;46(20):e00419. doi: 10.1002/marc.202500419 (PMC12536383; doi:10.1002/marc.202500419)
Supplement: Supplementary file 1 — Supporting file: marc70005‐sup‐0001‐SuppMat.pdf [file MARC-46-e00419-s001.pdf]

Supporting Information

Self-immolative Polyion Complexes

*Xueli Mei and Elizabeth R. Gillies**

X. Mei, E. R. Gillies
Department of Chemistry, The University of Western Ontario, London, Ontario, N6A 5B7, Canada
E-mail: egillie@uwo.ca

E. R. Gillies
Department of Chemical and Biochemical Engineering, The University of Western Ontario, London, Ontario, N6A 5B9, Canada

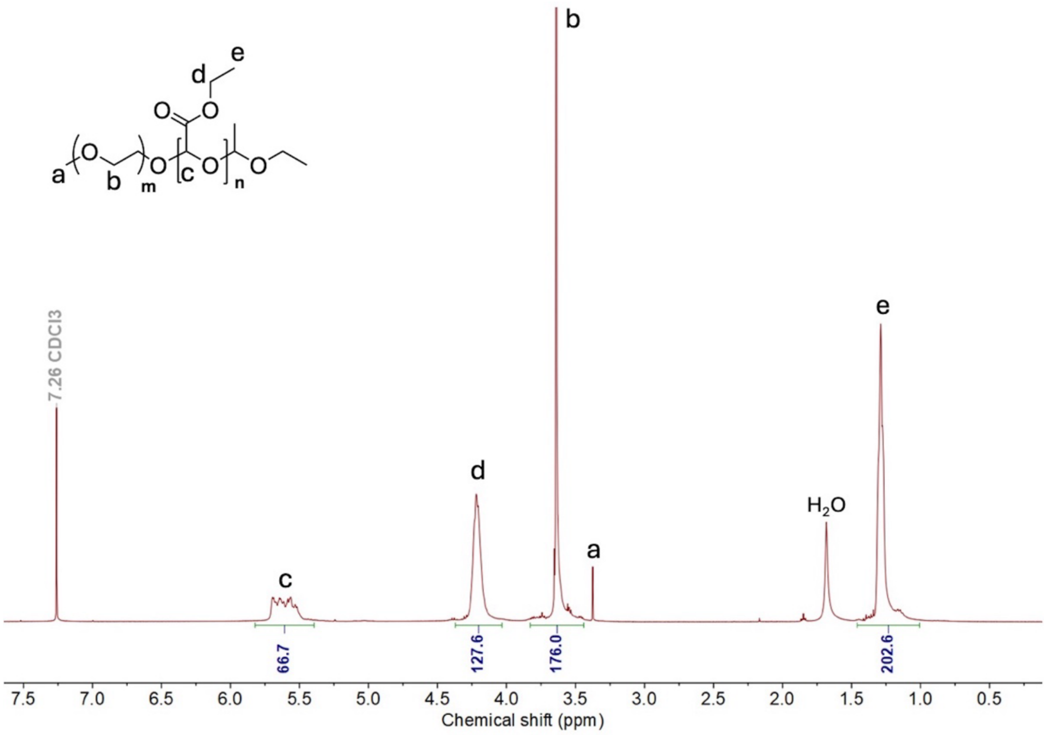

Figure S1. ^{1}H NMR spectrum of PEG-PEtG ($CDCl_3$, 400 MHz). Note that end-cap peaks are not clearly observed due to overlap with polymer peaks.

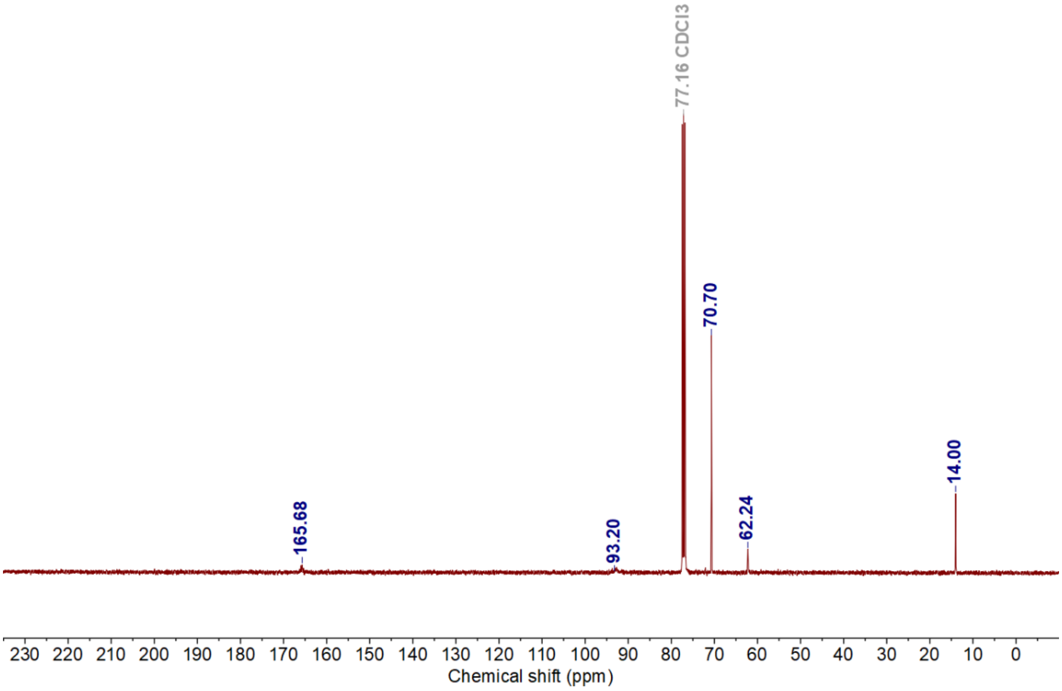

Figure S2. ^{13}C NMR spectrum of PEG-PEtG ($CDCl_3$, 100 MHz). Note that end-cap peaks are not observed due to the high degree of polymerization.

a)

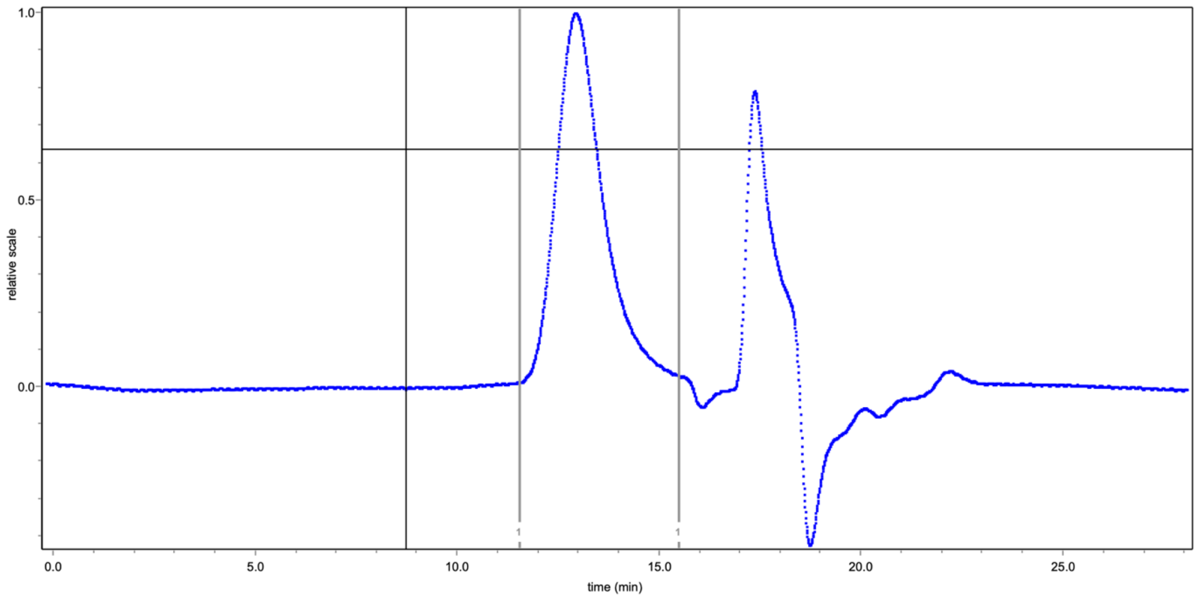

b)

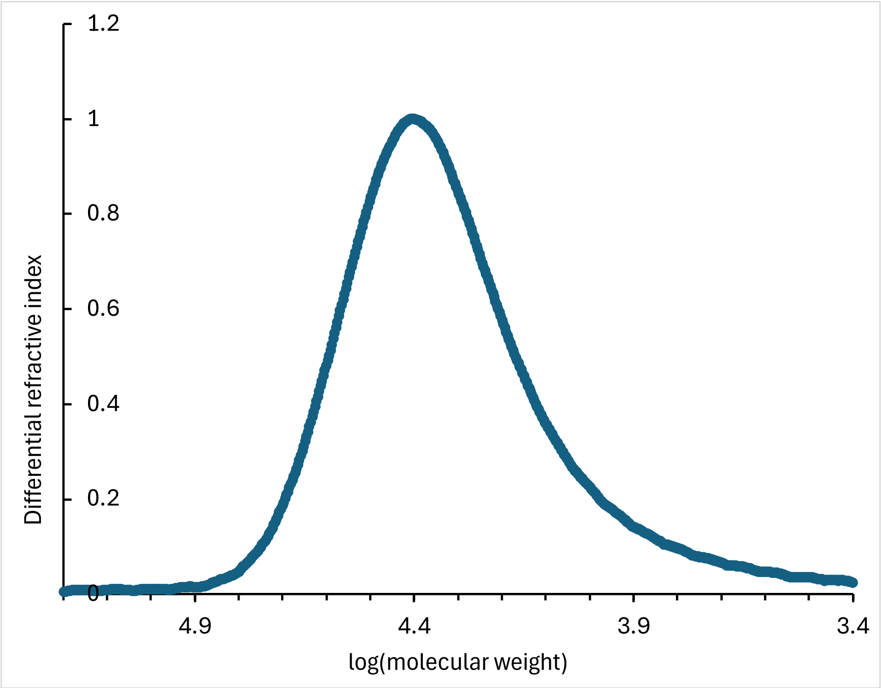

Figure S3. SEC trace of PEG-PEtG, run in DMF: a) Full trace; b) log(molecular weight) plot for the polymer peak relative to PMMA standards. Additional peaks in part a correspond to the solvent system.

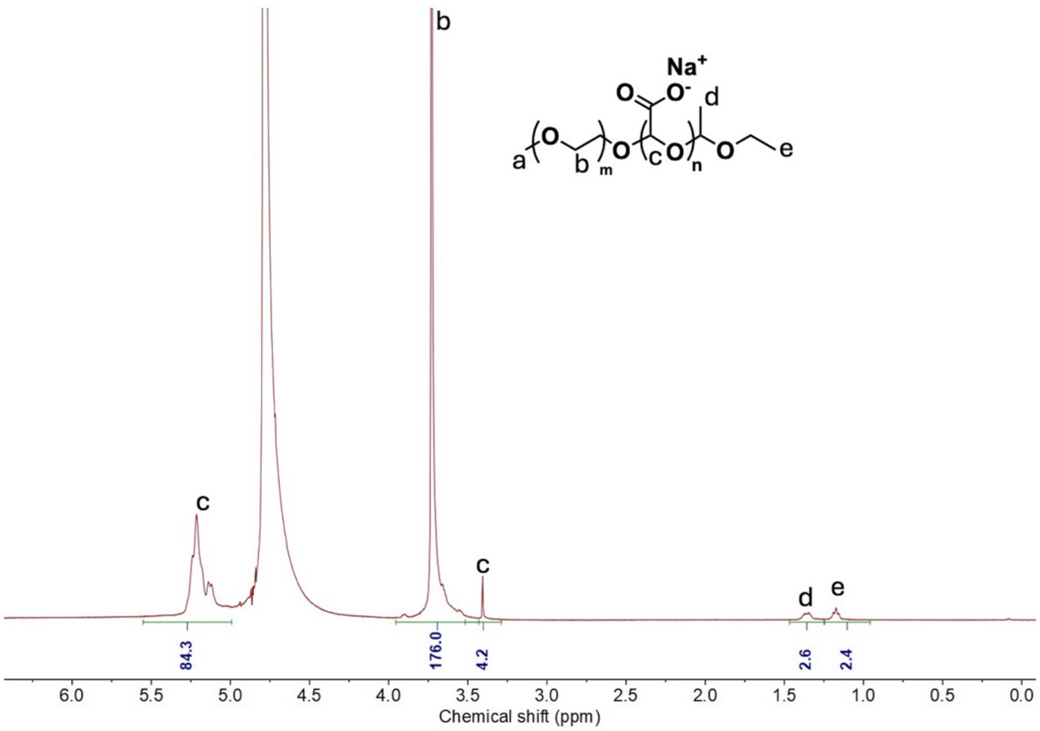

Figure S4. ^{1}H NMR spectrum of PEG-PGA (D_2O, 400 MHz).

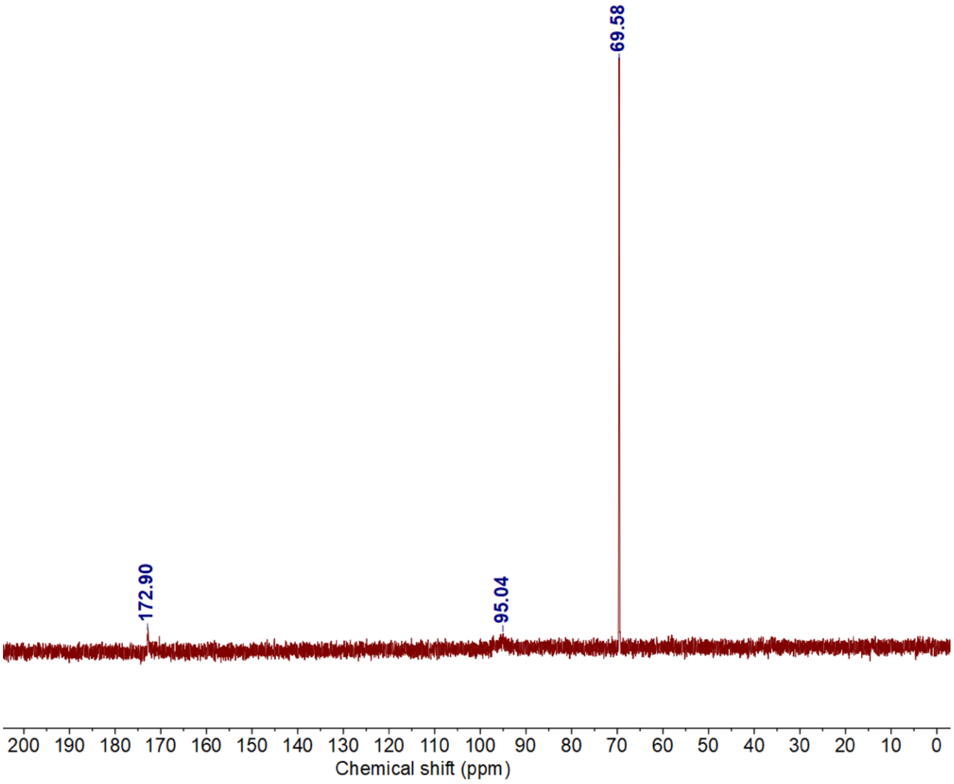

Figure S5. ^{13}C NMR spectrum of PEG-PGA (D_2O, 100 MHz). Note that end-cap peaks are not observed due to the relatively high degree of polymerization.

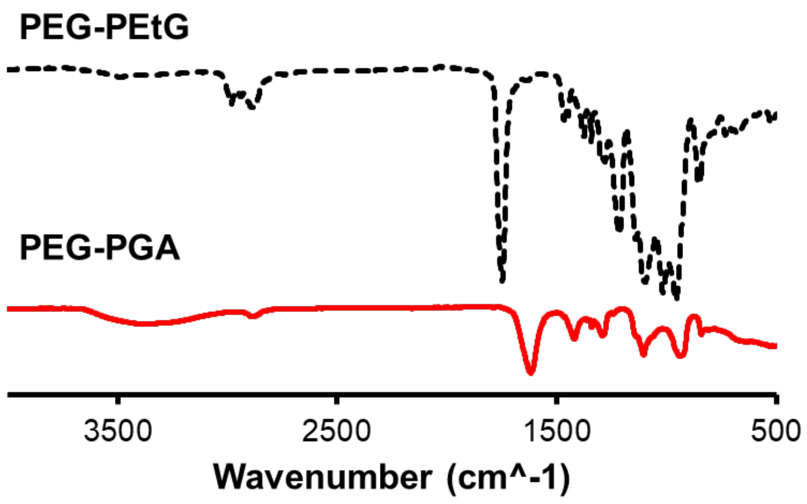

Figure S6. IR spectra of PEG-PGA (sodium salt) compared with the starting polymer PEG-PEtG showing the disappearance of the carbonyl peak of the ester group of PEG-PEtG around 1750 cm^{-1} and the appearance of a new carbonyl stretch of the carboxylate at approximately 1620 cm^{-1}.

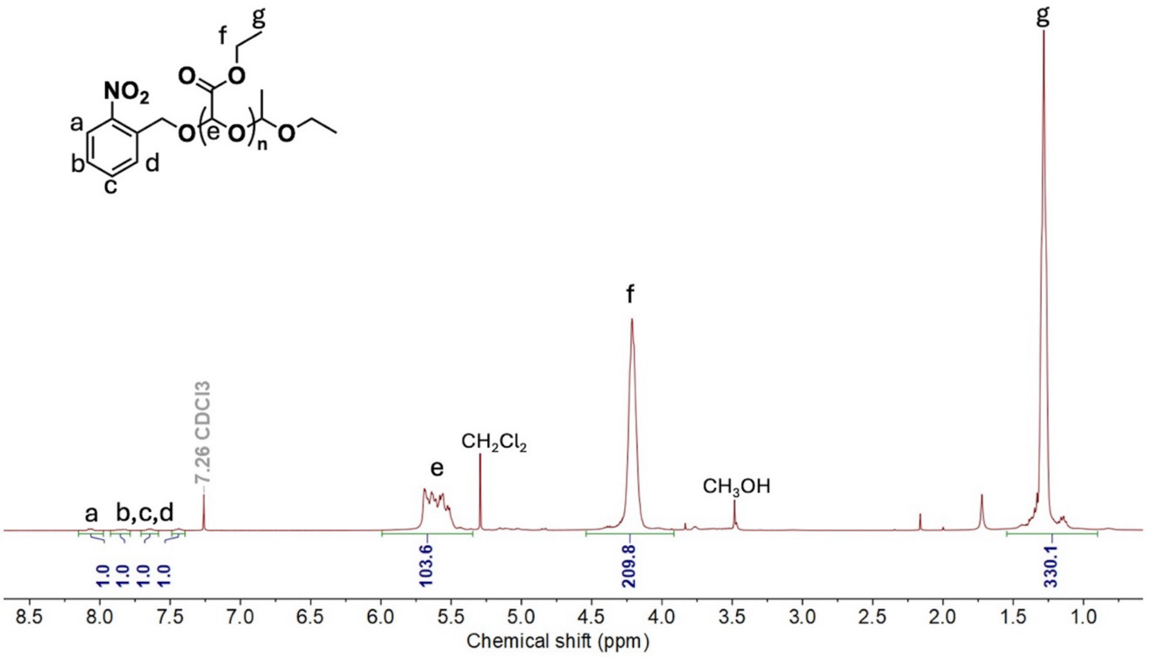

Figure S7. ^{1}H NMR spectrum of NB-PEtG ($CDCl_3$, 400 MHz). Note that some end-cap peaks are not clearly observed due to overlap with the polymer peaks.

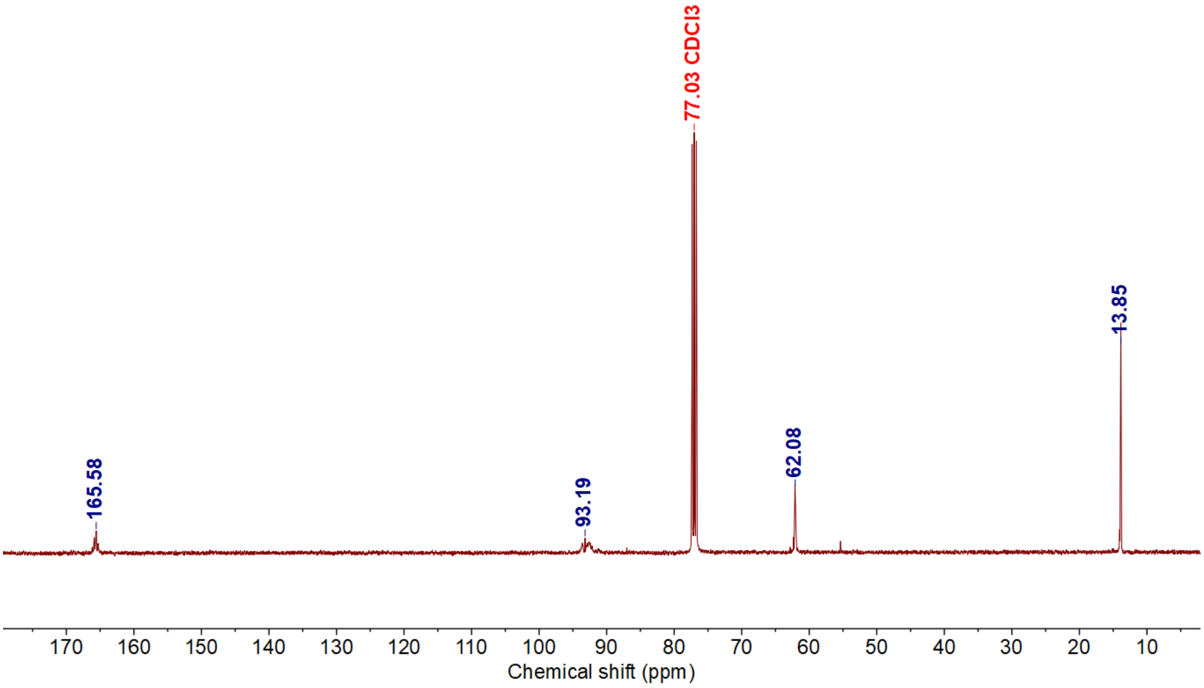

Figure S8. ^{13}C NMR spectrum of newly synthesized NB-PEtG ($CDCl_3$, 100 MHz). Note that end-cap peaks are not observed due to the relatively high degree of polymerization.

a)

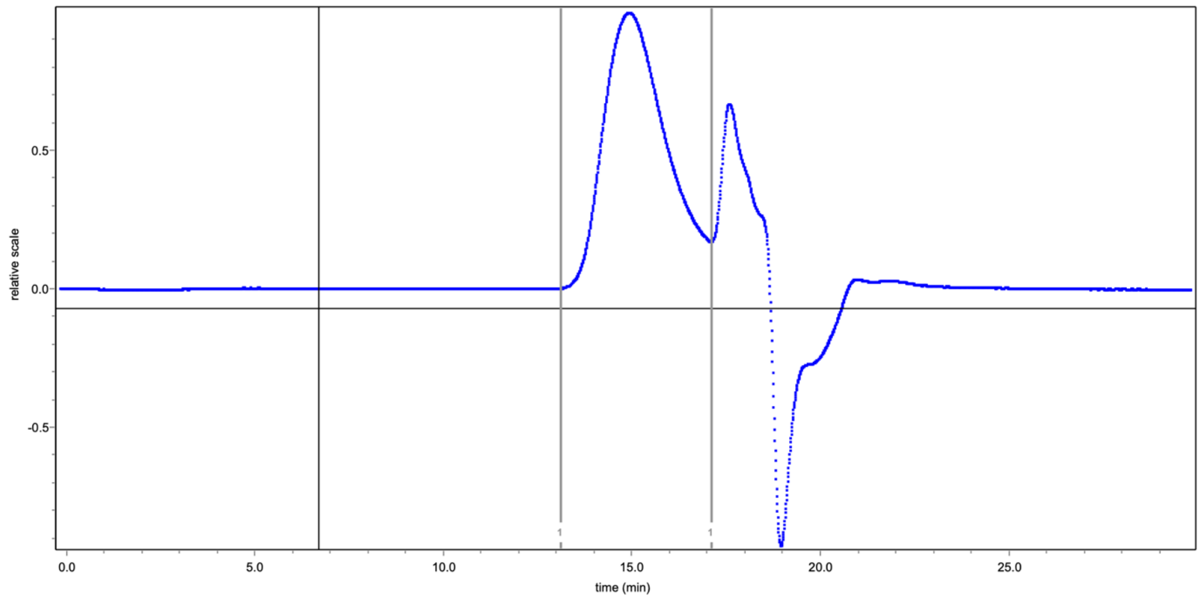

b)

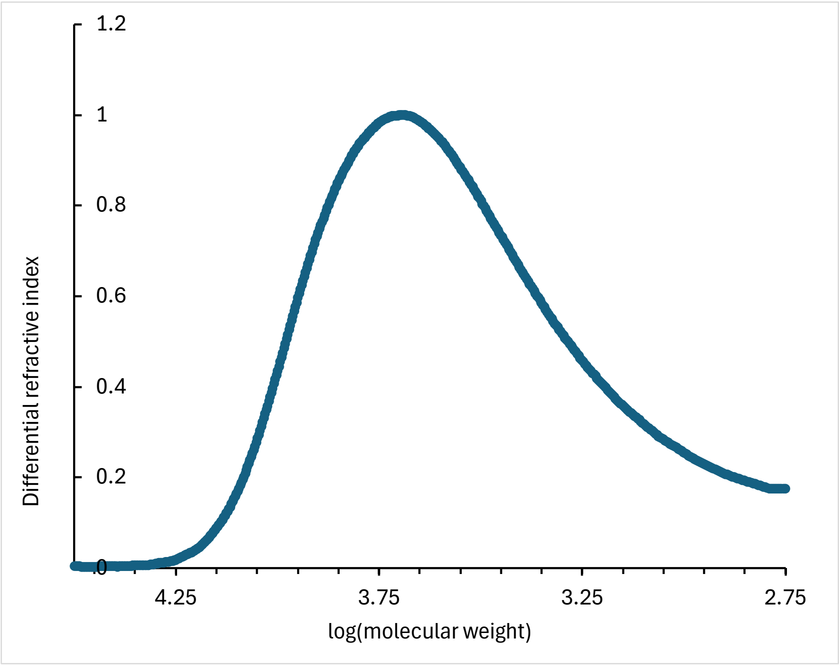

Figure S9. SEC trace of NB-PEtG, run in DMF: a) Full trace; b) log(molecular weight) plot for the polymer peak relative to PMMA standards. Additional peaks in part a correspond to the solvent system.

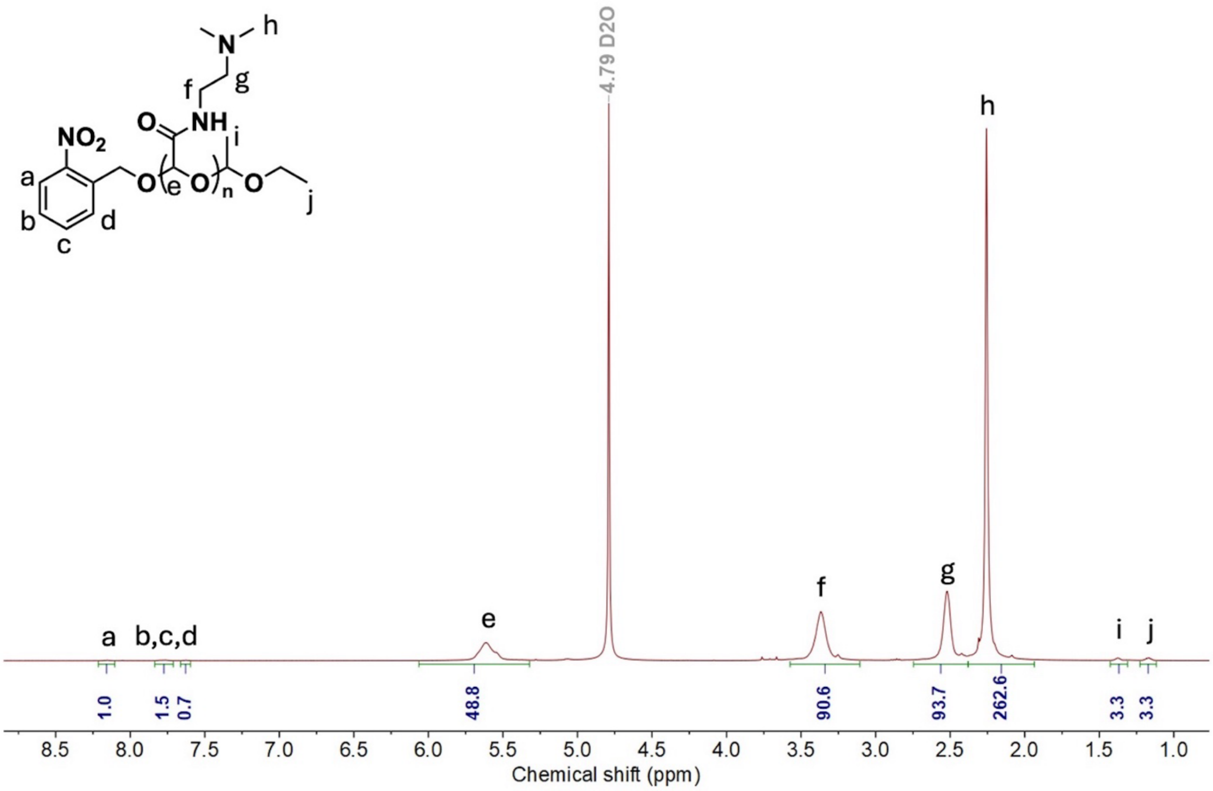

Figure S10. ^{1}H NMR spectrum of NB-PGAm (D_2O, 400 MHz).

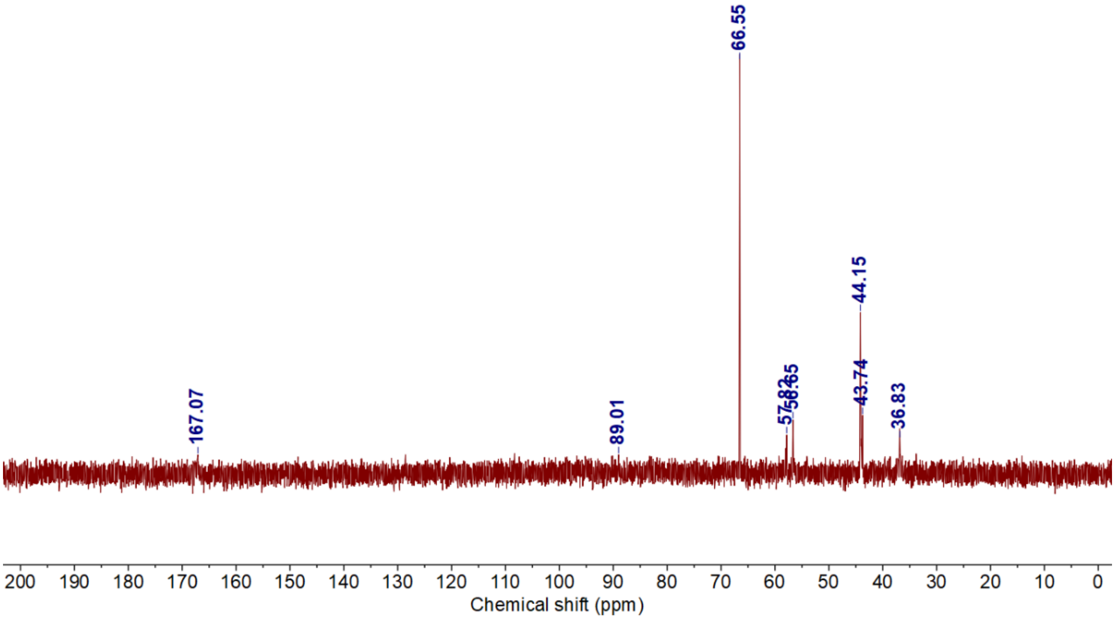

Figure S11. ^{13}C NMR spectrum of newly synthesized NB-PGAm (D_2O, 100 MHz). Note that end-cap peaks are not observed due to the relatively high degree of polymerization.

a)

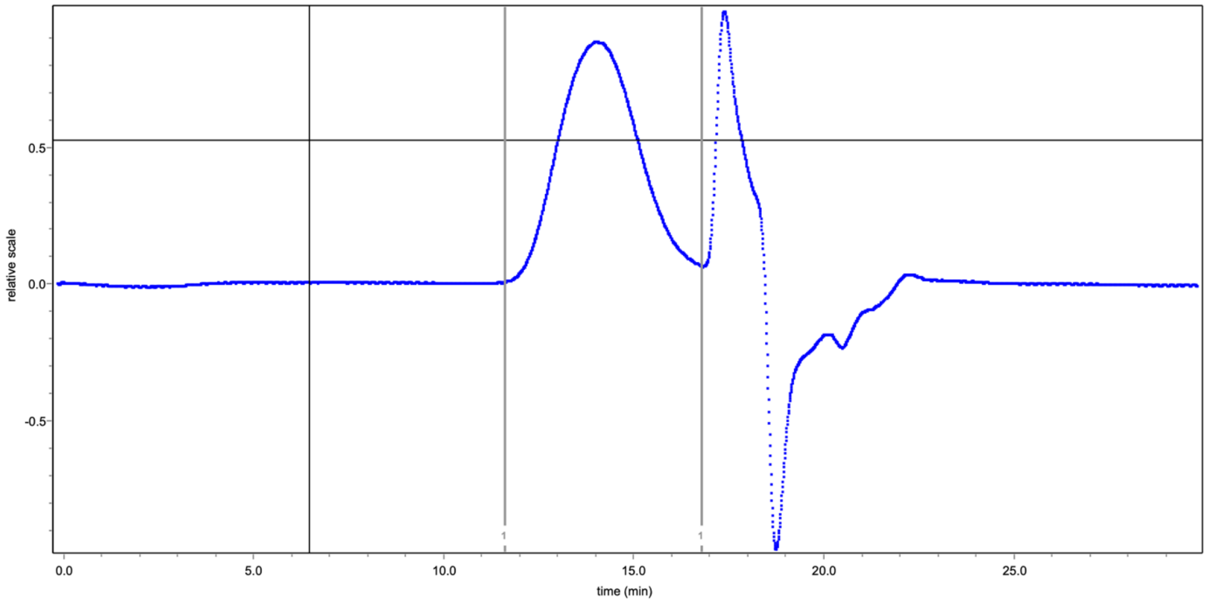

b)

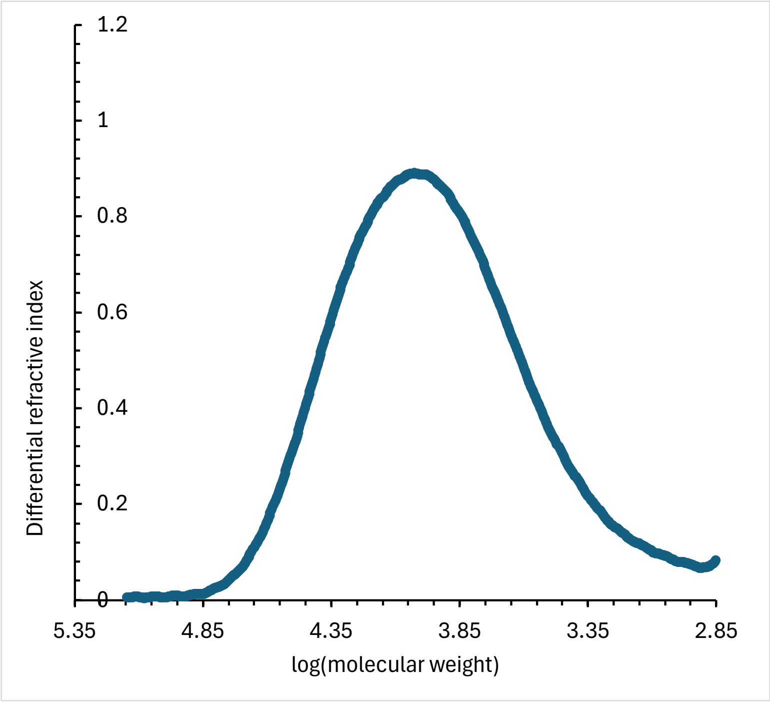

Figure S12. SEC trace of NB-PGAm, run in DMF: a) Full trace; b) log(molecular weight) plot for the polymer peak relative to PMMA standards. Additional peaks in part a correspond to the solvent system.

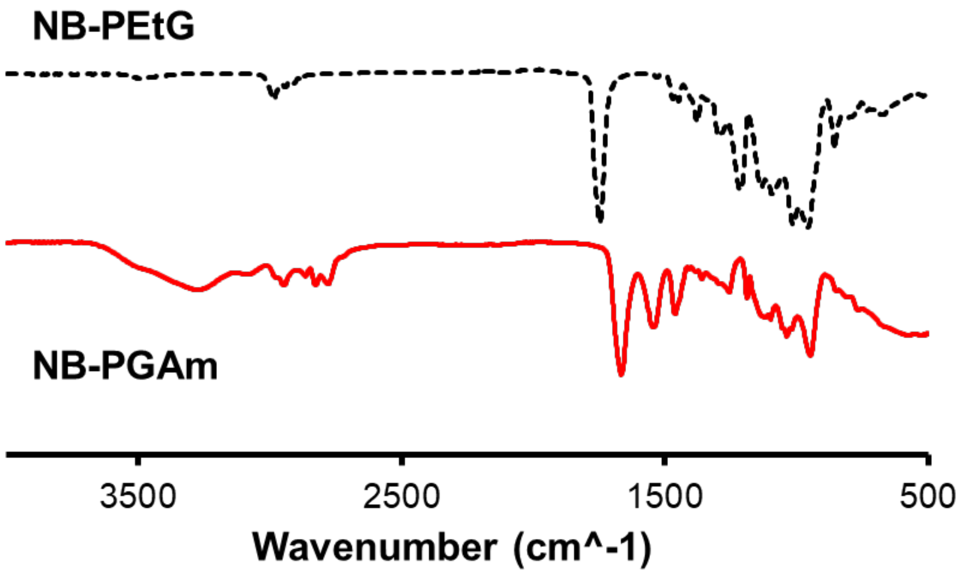

Figure S13. FT-IR trace of NB-PGAm compared with the starting polymer NB-PEtG, showing the disappearance of the carbonyl stretching peak of the ester group of NB-PEtG around 1750 cm^{-1} and a new -C=O stretch peak around 1660 cm^{-1} together with a new -NH stretch peak around 3280 cm^{-1} corresponding to the new amide group in NB-PGAm.

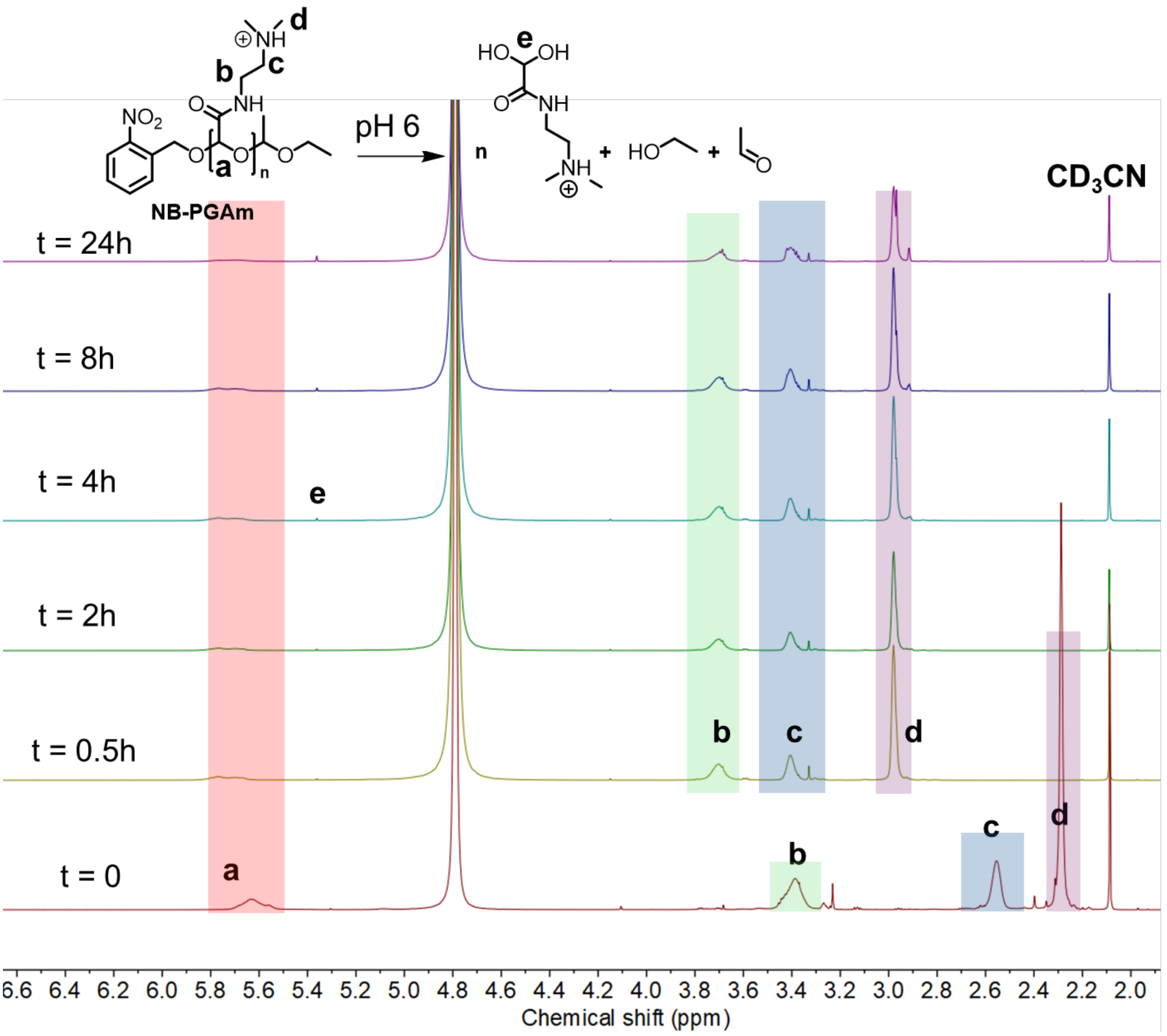

Figure S14. Overlay of ^{1}H NMR spectra of NB-PGAm over time at 37 °C in deuterated PBS (400 MHz). The pH was adjusted from 7.4 to 6 at t = 0.5 h. Low levels of depolymerization occurred, as evidenced by the appearance of the small peak e, corresponding to the depolymerization product *N*-[2-(dimethylamino)ethyl]-2,2-dihydroxyacetamide.

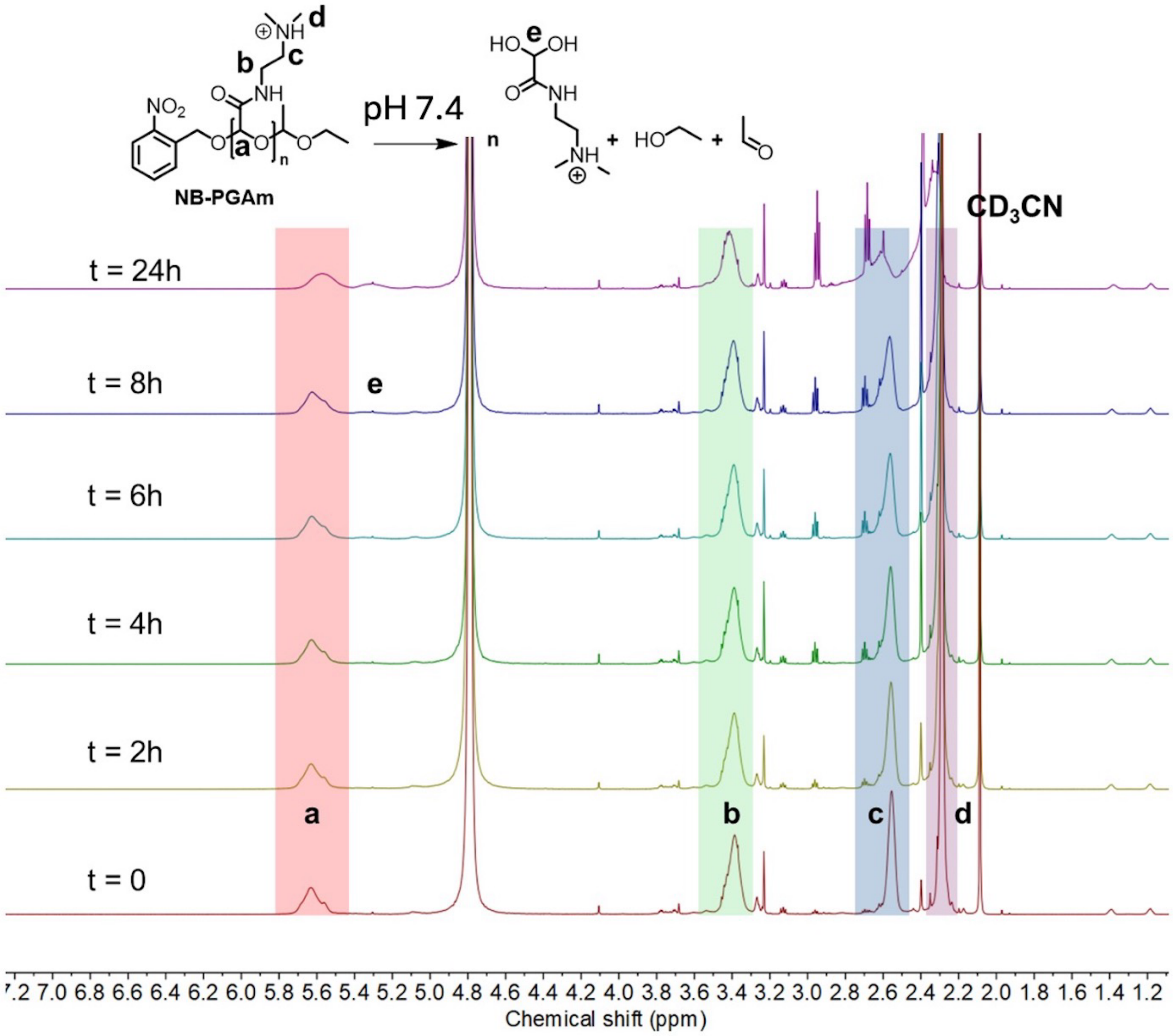

Figure S15. Overlay of ^{1}H NMR spectra of NB-PGAm over time at 37 °C in deuterated pH 7.4 PBS (400 MHz). Very low levels of depolymerization occurred as evidenced by the appearance of a very small peak e, corresponding to the depolymerization product *N*-[2-(dimethylamino)ethyl]-2,2-dihydroxyacetamide.

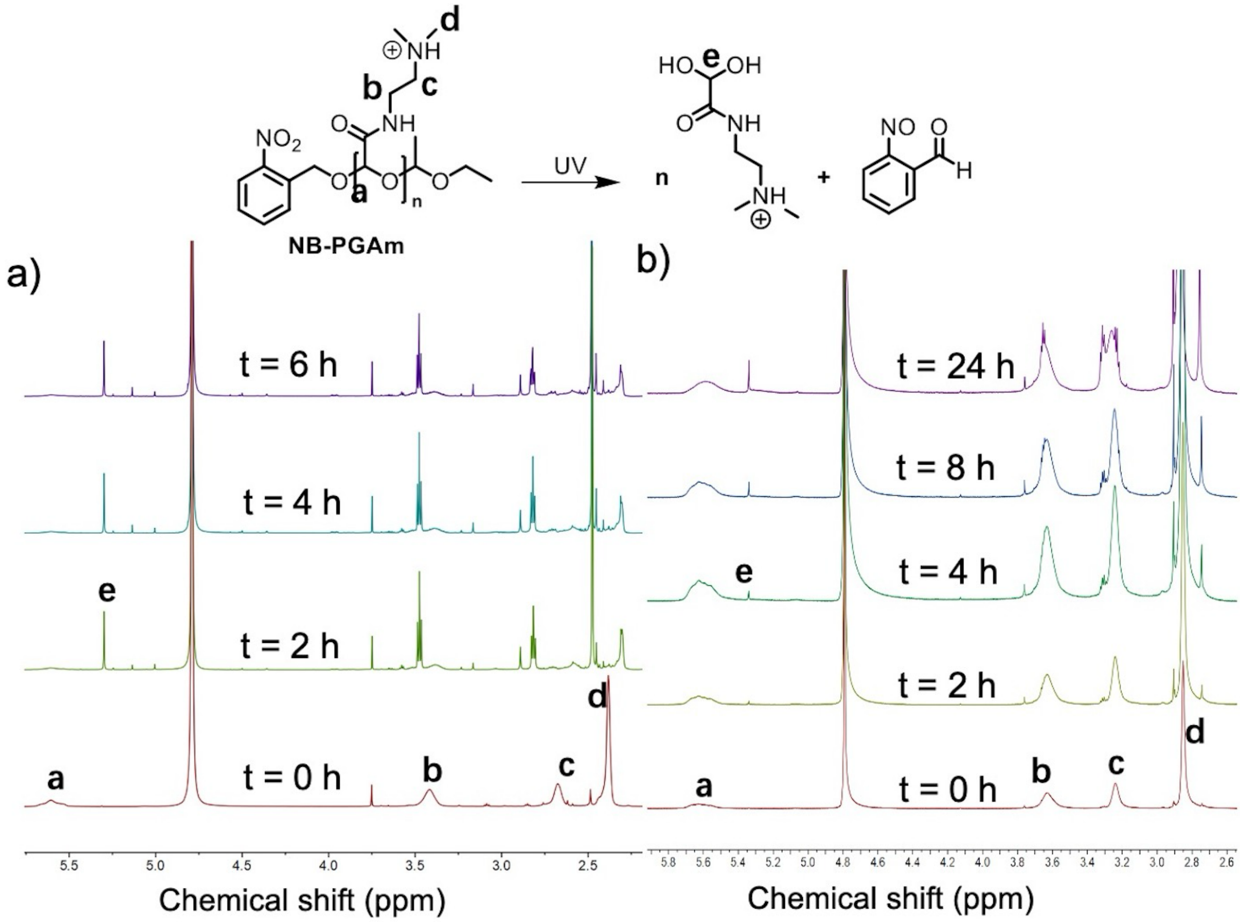

Figure S16. ^{1}H NMR spectral overlay of NB-PGAm over time at 20 °C in deuterated pH 7.4 PBS (400 MHz): a) before (t = 0) and after different periods of UV light irradiation; b) control sample kept in dark. With UV light irradiation, depolymerization occurred as evidenced by the disappearance of peak a and the appearance of peak e, corresponding to the degradation product. Minimal depolymerization was observed for the sample kept at dark, likely via gradual acetal end-cap hydrolysis instead.

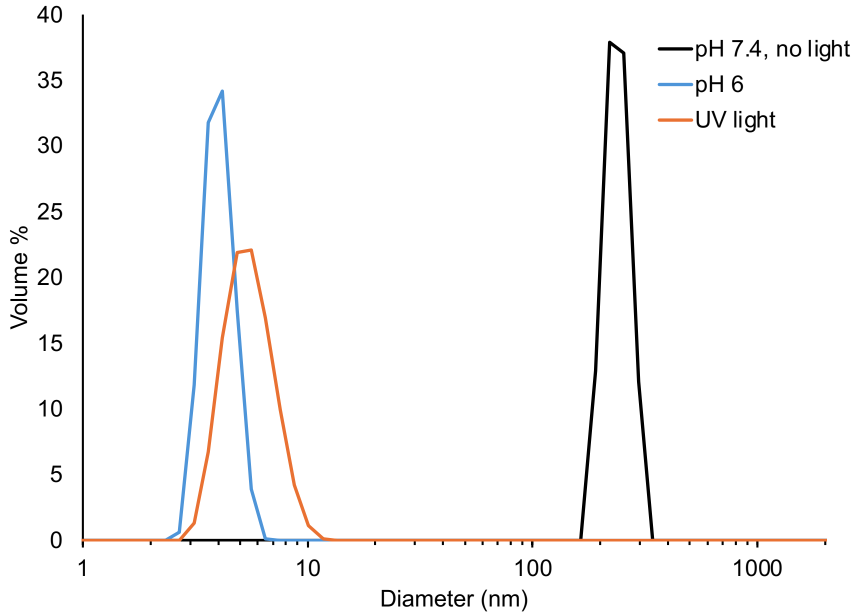

Figure S17. DLS volume distributions for PICs incubated at 37 °C at either pH 7.4 or 6 or with UV light irradiation (20 °C) after 72 h.

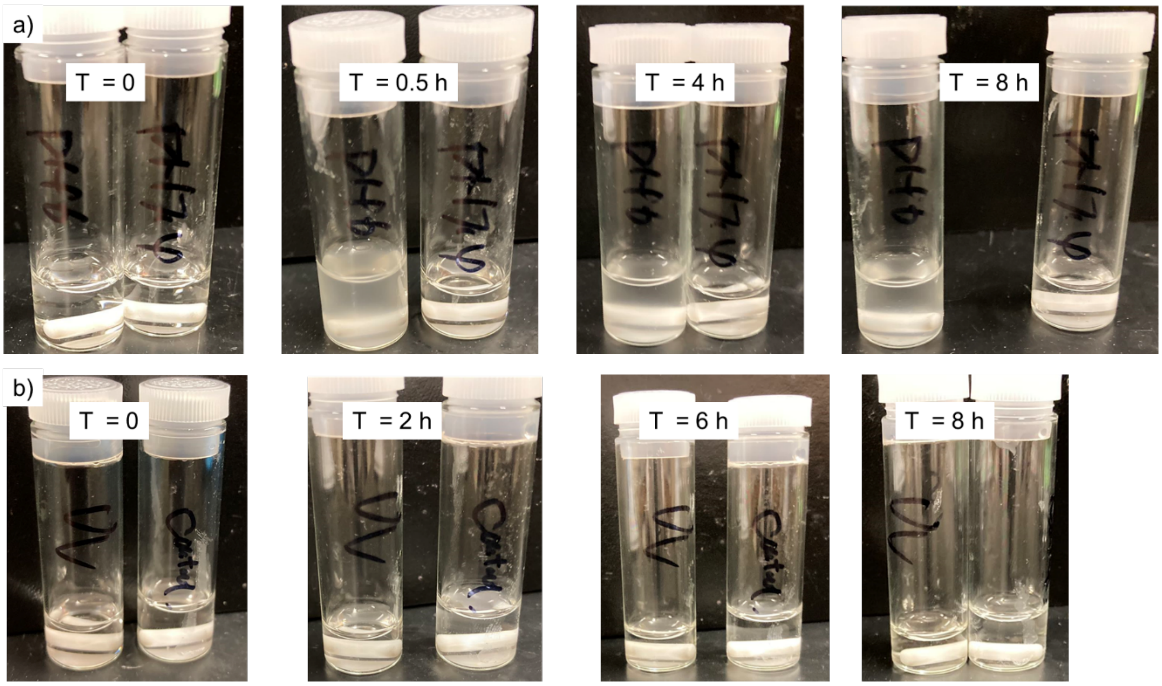

Figure S18. Pictures of PICs at different time points during the degradation study a) left: pH 6, right: pH 7.4 (control) and b) left: UV irradiated, right: kept at dark (control).

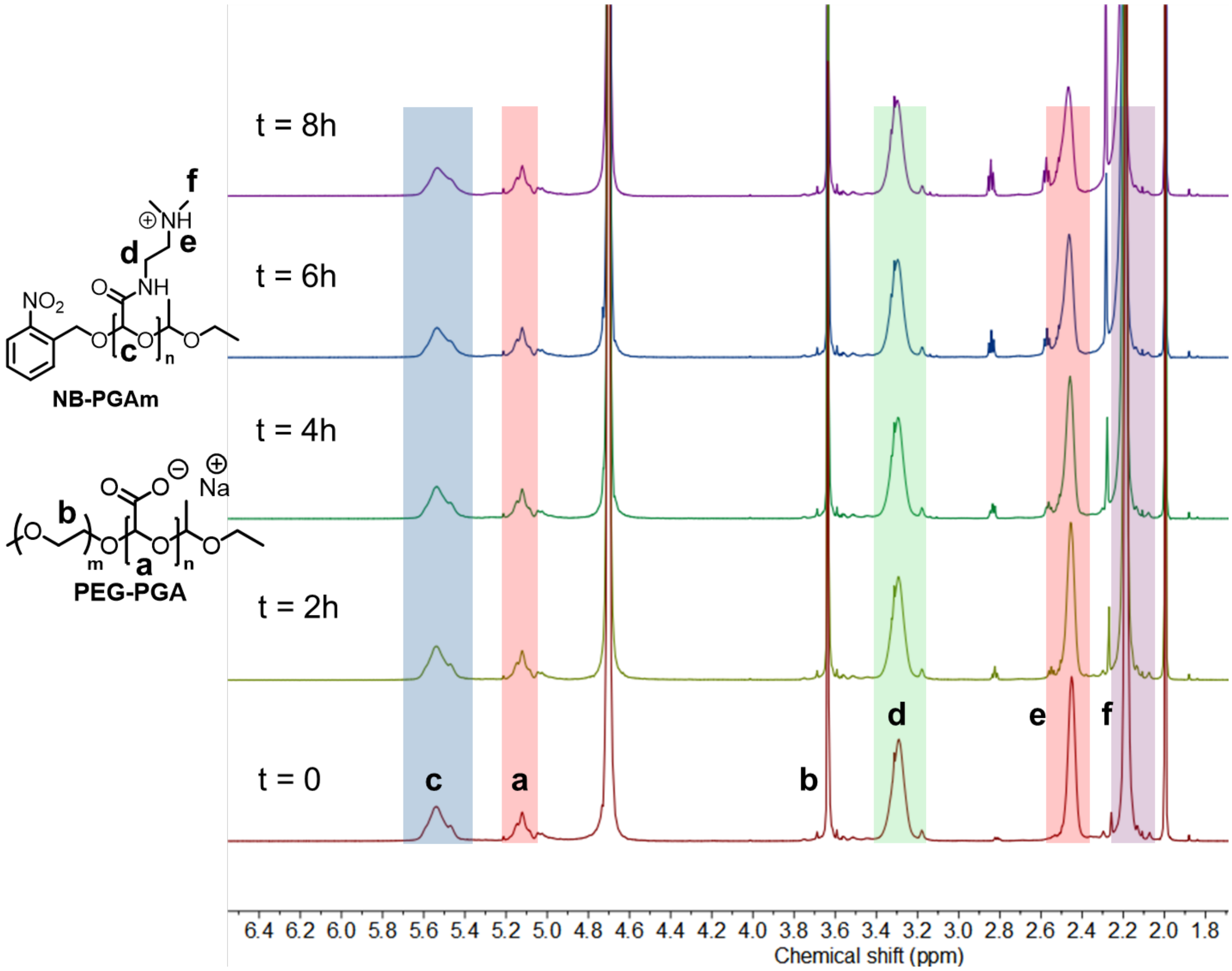

Figure S19. ^{1}H NMR spectral overlay of a PIC solution prepared at 0.6 anion:cation ratio at 10 mg/mL in deuterated PBS (pH 7.4) over time at 37 °C without adjusting the pH. Minimal depolymerization was observed for both polymers as the backbone peaks a and c remained intact.

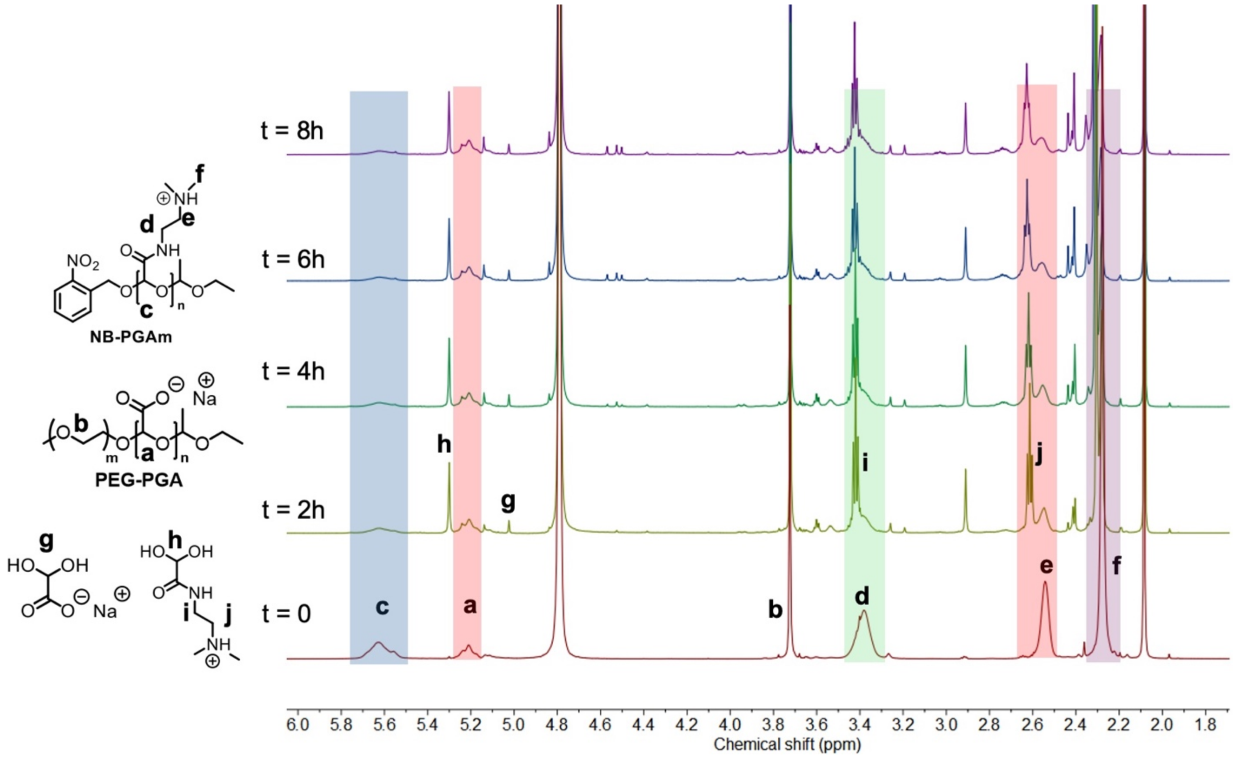

Figure S20. ^{1}H NMR spectral (400 MHz) overlay of PICs prepared at 0.6 anion:cation mixing ratio in deuterated PBS (pH 7.4) containing 0.1% acetonitrile before (t = 0) and after various time periods of irradiation with UV light. UV light led to greatly accelerated depolymerization of NB-PGAm, as indicated by the appearance of their corresponding small molecule depolymerization products, whereas the PEG-PGA only depolymerized to a small extent due to acetal end-cap cleavage.

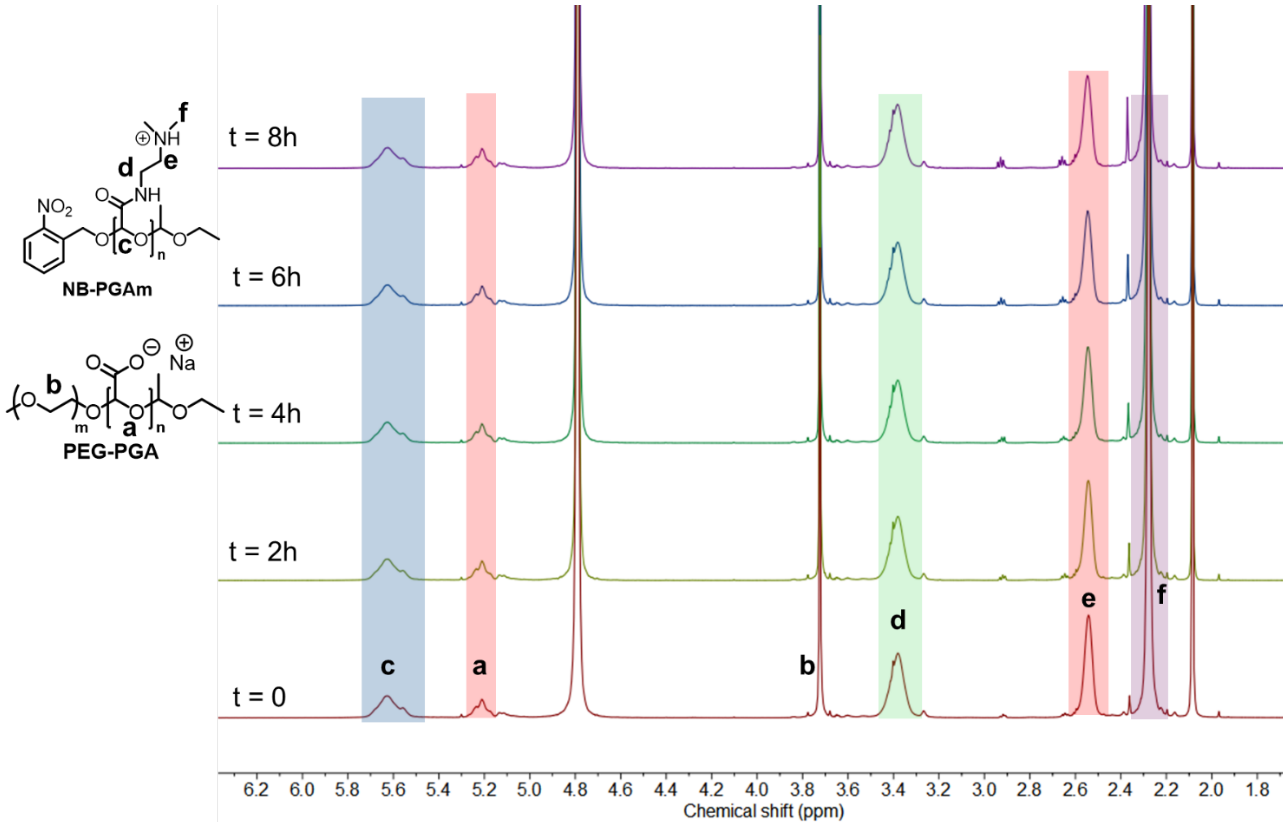

Figure S21. ^{1}H NMR spectral overlay of a PIC solution prepared at 0.6 anion:cation ratio at 10 mg/mL in deuterated PBS (pH 7.4) over time kept at 20 °C in the dark. Minimal depolymerization was observed for both polymers as the backbone peaks a and c remained intact.
